# Rare and misincorporated DNA N^6^-methyladenine is a hallmark of cytotoxic stresses for selectively stimulating the stemness and proliferation of glioblastoma cells

**DOI:** 10.1038/s41421-022-00399-x

**Published:** 2022-04-30

**Authors:** Cong Lyu, Yamei Niu, Weiyi Lai, Yu Wang, Yaning Wang, Peibin Dai, Chunhui Ma, Shaokun Chen, Yao Li, Guibin Jiang, Zhiyong Liang, Wenbin Ma, Zhengliang Gao, Wei-Min Tong, Hailin Wang

**Affiliations:** 1 The State Key Laboratory of Environmental Chemistry and Ecotoxicology, Research Center for Eco-Environmental Sciences, Chinese Academy of Sciences, Beijing, China; 2 University of Chinese Academy of Sciences, Beijing, China; 3 Department of Pathology, Institute of Basic Medical Sciences, Chinese Academy of Medical Sciences, School of Basic Medicine, Peking Union Medical College, Beijing, China; 4 Department of Neurosurgery, Peking Union Medical College Hospital, Chinese Academy of Medical Sciences and Peking Union Medical College, Beijing, China; 5 Yangzhi Rehabilitation Hospital (Shanghai Sunshine Rehabilitation Center), Tongji University School of Medicine, Shanghai, China; 6 Department of neurosurgery, Tongji Hospital, Tongji University School of Medicine, Shanghai, China; 7 Department of Pathology, State Key Laboratory of Complex Severe and Rare Disease, Molecular Pathology Research Center, Peking Union Medical College Hospital, Chinese Academy of Medical Sciences and Peking Union Medical College, Beijing, China

**Keywords:** DNA methylation, Tumour biomarkers

## Abstract

The entity of DNA N^6^-methyladenine (6mA) in mammals remains elusive and subsequently its roles in diseases are poorly understood. Here we exploited a bacterial DNA contamination-free and ultrasensitive UHPLC-MS/MS assay to reassess DNA 6mA in human glioblastomas and unveiled that DNA 6mA (~0.08 ppm) is extremely rare. By the use of two independent heavy stable isotope-labeling strategies, we further prove that the observed 6mA is solely generated by DNA polymerase-mediated misinocorporation. In vitro experiments point toward that the generation of misincorporated DNA 6mA is associated with the cellular stresses-caused release of RNA N^6^-methyladenine (m^6^A) nucleoside, which is profoundly inhibited by hypoxia milieu. Consistently, compared with normal brain tissues, DNA 6mA decreases in hypoxic human gliomas. Our data also strongly support that rare DNA 6mA rather than relatively abundant DNA 5-methylcytosine and 5-hydroxymethylcytosine is a hallmark of poor prognosis of *IDH1/2* mutation-absent glioblastoma patients, reflecting the incidence of cytotoxic stresses and subsequent release of m^6^A nucleoside. The released m^6^A nucleoside may selectively preserve a subset of the glioblastoma cells and stimulate their stemness and proliferation. Noteworthily, demethylation-inhibiting *IDH1* mutation increases the DNA 6mA content in human gliomas, but the depletion of the demethylase candidate ALKBH1 fails to do so, together suggesting the presence of other unknown 6mA demethylase for erasing misincorporated DNA 6mA. This is the first report on the identification of the misincorporated 6mA together with its origin and roles in diseases.

## Introduction

DNA N^6^-methyladenine (6mA) was identified in prokaryotes^1^ and unicellular eukaryotes^2–5^ four or five decades ago, and recently re-discovered in high eukaryotes^6–11^. This re-discovered epigenetic DNA modification is proposed to be implicated in a few diseases, e.g., gastric and liver cancer^12^ and glioblastoma^13^. In these diseases, the abundance of 6mA was shown to be high (~100–1000 ppm)^12,13^. However, the entity of post-replicative and epigenetic DNA 6mA remains as an active debate^14–16^. Bacterial DNA carrying overwhelming 6mA could contaminate cellular DNA^14–16^, causing 6mA overestimation even by most reliable liquid chromatography-tandem mass spectrometry (LC-MS/MS) assay^17^. Meanwhile, for current genome-wide DNA sequencing technologies, a few confounding factors together contribute to artificial or biased DNA 6mA peak calling^18,19^.

Instead of being post-replicative DNA modification, by the use of heavy stable isotope labeling, we and Musheev et al. independently showed the presence of non-epigenetic and misincorporated DNA 6mA in mammalian genomes^20,21^. We showed that the depletion of potential methylase Mettl4 and demethylase Alkbh1^10,22^ do not alter the level of the identified misincorporated DNA 6mA^20^. Despite of being an erroneous product of DNA amplification, the roles of the misincorporated 6mA are not explored in diseases. On the other hand, Xie et al.^13^ demonstrate the extraordinary enrichment of DNA 6mA in the genome of glioblastoma. Regarding the intensive controversy on mammalian DNA 6mA, it is yet worth corroborating the entity and identity of DNA 6mA in glioblastoma.

To address above issues, by combining two independent heavy stable isotope tracing strategies with bacterial DNA contamination-free and ultrasensitive ultra-high-performance LC-MS/MS (UHPLC-MS/MS) technology, we reassessed DNA 6mA in human gliomas, including primary and culturing glioblastoma cells, and for the first time unveiled the origin and roles of DNA 6mA in human gliomas and potential underlying mechanisms.

## Results

### Bacterial DNA contamination-free and ultrasensitive UHPLC-MS/MS detection unveils extremely rare DNA N^6^-methyladenine in human glioma

**Fig. 1:**
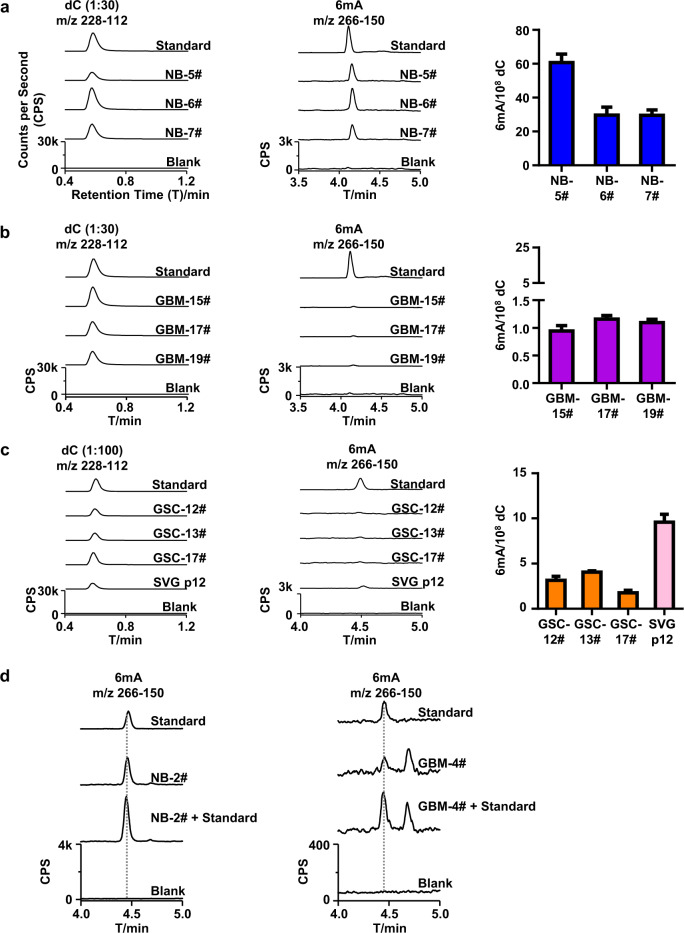
Identification of DNA 6mA in human normal brain tissues and glioblastomas. **a**–**c** UHPLC-MS/MS chromatograms and quantification of DNA dC and 6mA in normal brain (NB) tissues (**a**), Glioblastoma (GBM) (**b**), and Glioblastoma Stem Cells (GSCs) and glial cell line (SVG p12) (**c**). **d** UHPLC-MS/MS chromatograms of DNA 6mA in the genome of control brain tissues (NB-2#, ~22 6mA per 10^8^ dC) spiked with or without 6mA standard (~13 6mA per 10^8^ dC) and in the genome of GBM-4# (~1.2 6mA per 10^8^ dC)) spiked with or without 6mA standard (~1.2 6mA per 10^8^ dC). Blank: ultrapure water underwent the purification and digestion as same as real samples.

In order to reassess DNA N^6^-methyladenine (6mA) in human glioma, we first improved both our DNA extraction protocol and UHPLC-MS/MS assay. For this purpose, all reagents used for DNA extraction were pretreated using a cartridge filled with beads adsorbing both DNA and free 6mA nucleosides. By this pretreatment, the possible contamination of bacteria DNA can be completely removed. Meanwhile, a UHPLC-MS/MS system must be maintained to be clean throughout the detection. To guarantee the cleanup of the UHPLC-MS/MS system, pure water was injected and no artificial 6mA peak must be observed. Benefiting from the cartridge pretreatment and the clean UHPLC-MS/MS system, indeed we did not see any 6mA signal in the blank (Fig. 1), which involved DNA extraction procedure and underwent full set of UHPLC-MS/MS assay. An excellent linearity for 6mA (correlation coefficient: *R*^2^ ≥ 0.999) was obtained with a dynamic range of 10−2000 × 10^–18 ^mol (or 10–2000 amol) (Supplementary Fig. S1a). To estimate the limit of the detection (LOD) of 6mA in the presence of genomic DNA background of glioma cells, different amounts of 6mA standard (0.1 pM – 2 pM) were spiked with genomic DNA of glioma cells. By the injection of 10 μL spiked genomic DNA solution (~1.1 μg genomic DNA), the LOD of 6mA was estimated to be ~10 amol (Equivalent: ~0.98 6mA per 10^8^ dC) (Supplementary Fig. S1b), which was 50-fold lower than previous reports showing a LOD of ~500 amol^10,21,23^, suggesting a 50-fold enhanced sensitivity.

Armed with bacterial DNA contamination-free and ultrasensitive UHPLC-MS/MS assay, we measured three normal brain tissue samples (NB), three human glioblastoma (GBM, grade IV) samples and three patient-derived glioblastoma stem cells (GSCs). Unexpectedly, we observed extremely low 6mA in all the tested samples, including NBs (~30–60 6mA per 10^8^ dC, Fig. 1a), GBMs (~1.0–1.5 6mA per 10^8^ dC, Fig. 1b) and GSCs (~2.0–4.0 6mA per 10^8^ dC, Fig. 1c). Compared with NBs, GBMs and GSCs displayed much lower DNA 6mA content. Evidently, the levels of DNA 6mA in GBMs and GSCs detected by us are approximately four orders of magnitude lower than that as reported by Xie et al. (~1000 ppm, Eqv to 10^5^ 6mA per 10^8^ dC)^13^.

To verify the accuracy of the above detection, known amount of 6mA standard was spiked in the genomic DNA of one NB sample and one GBM sample. For the genome of normal brain tissue NB-2# (~22 6mA per 10^8^ dC), the 6mA was detected about ~37 6mA per 10^8^ dC when spiked with 6mA standard (13.0 6mA per 10^8^ dC); for the genome of GBM-4# (~1.2 6mA per 10^8^ dC), the 6mA content increased to (~2.7 6mA per 10^8^ dC) when spiked with 6mA standard (~1.2 6mA per 10^8^ dC (Fig. 1d). If we had underestimated the 6mA in the tested sample, we would not have observed the dramatic increase in 6mA peak by spiking with comparable 6mA standard. These results consistently support the accuracy of our UHPLC-MS/MS measurement.

Enlightened with above results, we also examined other types of human gliomas, Astrocytomas (As, grade II) and Anaplastic Astrocytomas (AAs, grade III). Consistently, both As (~7.0–20 6mA per 10^8^ dC, Supplementary Fig. S2a) and AAs (~1.0–6.0 6mA per 10^8^ dC, Supplementary Fig. S2b) displayed extremely low 6mA.

Collectively, these results strongly support the presence of extremely low 6mA in human gliomas, including As, AAs, GBMs, and GSCs.

### The generation of 6mA in glioma is independent of DNA methyltransferase

**Fig. 2:**
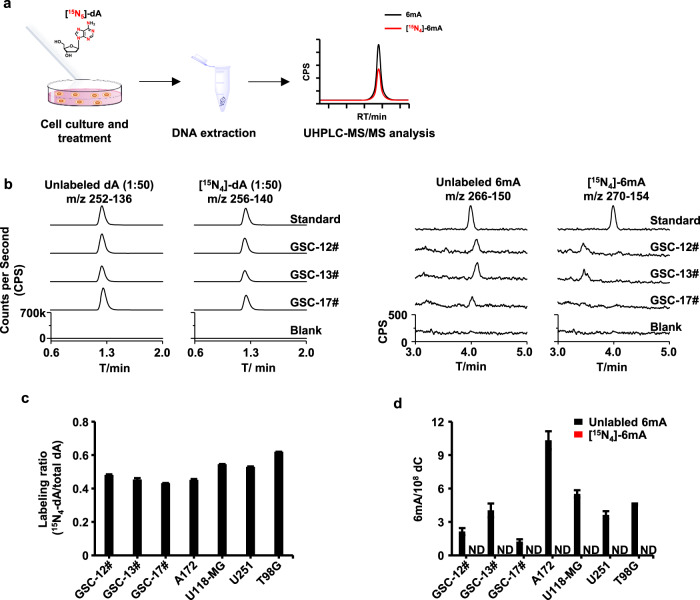
Characterization of DNA 6mA in GSCs and glioblastoma cell lines by heavy stable isotope tracer [15N5]-dA. **a** Flow diagram of tracing methylase-deposited DNA 6mA in glioma cells by [^15^N_5_]-dA. **b** UHPLC-MS/MS chromatograms of the labeled dA and 6mA in the genome of glioma stem cells (GSCs). **c**, **d** Quantification of the labeled dA (**c**) and 6mA (**d**) in the genomic DNA of human GSCs and human glioblastoma cell lines. ND, not detectable.

Next, by taking advantage of the unique heavy stable isotope-labeled adenosine tracing technology^14,20^, we investigated the origin of DNA 6mA in human gliomas. In brief, the initial tracer [^15^N_5_]-dA can be efficiently converted into [^15^N_4_]-dATP and incorporated into genomic DNA via DNA replication in mammalian cells^14^ (Fig. 2a). If there is any methyltransferase that can deposit a methyl group at the N6 atom of the labeled [^15^N_4_]-dA, [^15^N_4_]-6mA should be detected. As surrogate of human gliomas, we characterized three human GSCs (or primary glioblastomas) and four human glioblastoma cell lines (Fig. 2 and Supplementary Fig. S3). Approximately half of genomic dA was effectively labeled in the form of [^15^N_4_]-dA in the genomic DNAs (Fig. 2b, c and Supplementary Fig. S3a). However, we could not detect any [^15^N_4_]-6mA signal in the genomes of all tested GSCs (Fig. 2b, d) and glioblastoma cell lines (Supplementary Fig. S3b and Fig. 2d).

Moreover, we exploited second heavy stable isotope-labeling reagent [D_3_]-l-methionine to affirm our findings (Supplementary Fig. S4a). [D_3_]-l-methionine can be converted into stable isotope-labeled methyl donor S-adenosyl-l-methionine in cells^20^, and thus be utilized by potential DNA methyltransferase to generate DNA [D_3_]-6mA. However, as treated with [D_3_]-l-methionine, [D_3_]-6mA was not detected in the genomes of three GSCs (Supplementary Fig. S4c) and four glioblastoma cell lines (Supplementary Fig. S4e). In contrast, the labeling efficiency of 5mC of ~ 50% was labeled in almost all tested cell lines (Supplementary Fig. S4b, d). Of note, we did not detect any DNA [D_3_]-6mA in normal glial cells yet (SVG p12 cell line, Supplementary Fig. S4e).

The above results strongly support that the observed 6mA is generated in a DNA 6mA methylases-independent manner. Due to the lack of the action of 6mA methylases, the observed DNA 6mA is attributed to be caused by erroneous misincorporation in a DNA amplification manner. Although we cannot examine the origin of 6mA by direct assay of glioma tissues, these results obtained from the cultured glioblastoma cell lines and primary GSCs may suggest that the origin of the detected 6mA in glioma cells is independent of DNA methyltransferases. In other words, the observed DNA 6mA is associated with DNA amplification-caused misincorporation in gliomas.

### Global genomic DNA 6mA contents significantly decrease in glioma

**Fig. 3:**
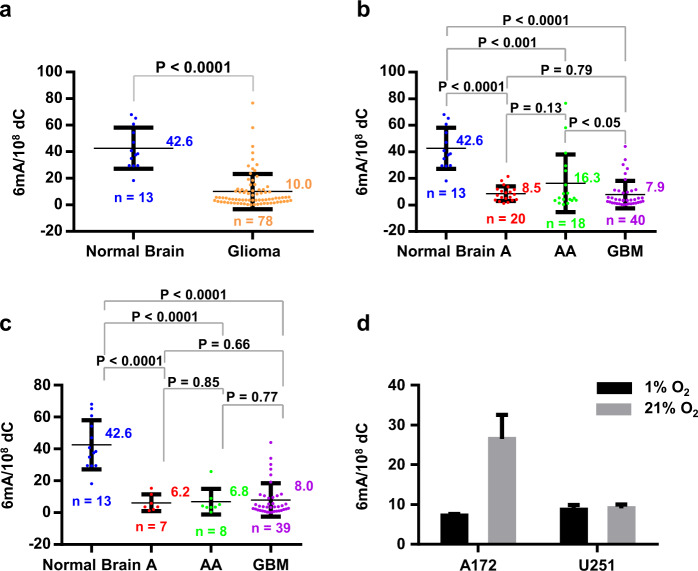
Genomic DNA 6mA content at global level significantly decreases in hypoxic human gliomas. **a** DNA 6mA content in glioma tissues. Normal brain tissues: *n* = 13; glioma tissues: *n* = 78. **b** DNA 6mA contents in glioma tissues and normal brain tissues. Normal brain tissues: *n* = 13; Astrocytoma (A) tissues: *n* = 20; Anaplastic Astrocytoma (AA) tissues: *n* = 18; Glioblastoma (GBM) tissues: *n* = 40. **c** DNA 6mA content in glioma tissues with IDH1^mut^ being excluded. Normal brain tissues: *n* = 13; A tissues: *n* = 7; AA tissues, *n* = 8; GBM tissues: *n* = 39. **d** Hypoxia reduces misincorporated DNA in cultured human glioblastoma cells. Statistical significance was determined by Student’s unpaired *t*-test.

We further investigated the roles of misincorporated DNA 6mA in human gliomas. For this purpose, we measured genomic 6mA in 13 normal brain (NB) tissues and 78 human glioma tissues (Table 1). The average values of DNA 6mA of normal brain and glioma tissues are about 42.6 6mA per 10^8^ dC and 10.0 6mA per 10^8^ dC (Fig. 3a), respectively. By statistical analysis, the 6mA content in glioma tissues significantly decreases compared to normal brain tissues (*P* < 0.0001) (Fig. 3a). In addition, we characterized DNA 6mA for one patient who first had astrocytoma (A-16#) and then secondary GBM (GBM-6#) (Table 1). The 6mA levels of A-16# and GBM-6# were ~21.5 6mA per 10^8^ dC and 3.7 6mA per 10^8^ dC (Supplementary Fig. S5a), respectively. The results confirmed the extremely low DNA 6mA even in malignant gliomas.

**Table 1:** Clinical characteristics of patients.

Clinical variable	(*n* = 78)
Age at diagnosis (years)	
Median	48.5
Range	23–76
Gender, no (%)	
Male	41 (52.5%)
Female	35 (44.9%)
N.A.	2 (2.6%)
Grade, no (%)	
II	20 (25.6%)
III	18 (23.0%)
IV	40 (51.3%)
Recurrence, no (%)	
No	75 (96.2%)
Yes	3 (3.8%)
Survival (months)	
Median	11
Range	< 1–76
IDH1 mutation status, no (%)	
Wild-type	54 (69.2%)
Mutated	22 (28.2%)
N.A.	2 (2.6%)

Next, to investigate the correlation between the statistical significance of 6mA content and the malignancy grade of glioma, we analyzed the statistical significance between A (grade II), AA (grade III), and GBM (grade IV) (Fig. 3b). The 6mA content significantly decreases in all classified glioma (*P* < 0.0001–0.001), including A, AA, and GBM, compared with normal brain tissues. Among three grades of glioma (A, AA, and GBM), there is only a significant decrease of 6 mA content in GBM (*n* = 40) compared with AA (*n* = 18) (*P* < 0.05). However, by excluding the patients with isocitrate dehydrogenase 1 (*IDH1*) mutation, we found that all types of glioma patients displayed much lower DNA 6mA content compared to the normal tissues and no statistic difference in between (Fig. 3c). The reason for excluding *IDH*1/2 mutation is given in the Section of increase of the misincorporated DNA 6 mA by *IDH1* mutation.

Since hypoxia is prevalent in glioma cells (including glioblastoma)^24^, we examined DNA 6 mA in glioblastoma cells under hypoxic culturing conditions. As expected, compared with normoxia (21% O_2_), hypoxia (1% O_2_) indeed reduced the misincorporated DNA 6mA content (Fig. 3d). This result suggests that hypoxia may profoundly reduce the misincorporated DNA 6 mA.

In view of the genome-wide DNA cytosine hypomethylation in cancer genomes^25^, we additionally detected the DNA 5-methylcytosine (5mC) content and also observed a significant decrease in human glioblastoma tissues compared to normal brain tissues (*P* < 0.0001) (Supplementary Fig. S6a). The average 5mC levels of normal brain and glioblastoma tissues are about 4.3 5mC per 100 dC and 3.4 5mC per 100 dC, respectively. However, there is no significant decrease in A and AA compared with normal brain tissues (Supplementary Fig. S6a). By a detailed statistical analysis according to the glioma malignancy classification, only GBM shows significant reduction in 5mC compared to any of normal brain tissues (*P* < 0.0001), A (*P* < 0.0001) or AA (*P* < 0.05) (Supplementary Fig. S6a). By excluding *IDH1* mutated-patients, both Anaplastic Astrocytomas (AAs) and GBMs display significant reduction in 5mC compared to normal brain tissues (Supplementary Fig. S6b). Consistently, the secondary GBM (GBM-6#) (Table 1) displays lower 5mC compared to first astrocytoma (A-16#) (Supplementary Fig. S5b).

Taken together, we demonstrated that the widespread genome-wide hypomethylation in cancer cells not only occurred in the canonical 5mC DNA modification but also existed in non-epigenetic and misincorporated DNA 6mA.

### Increase of misincorporated DNA 6mA by *IDH1* mutation

**Fig. 4:**
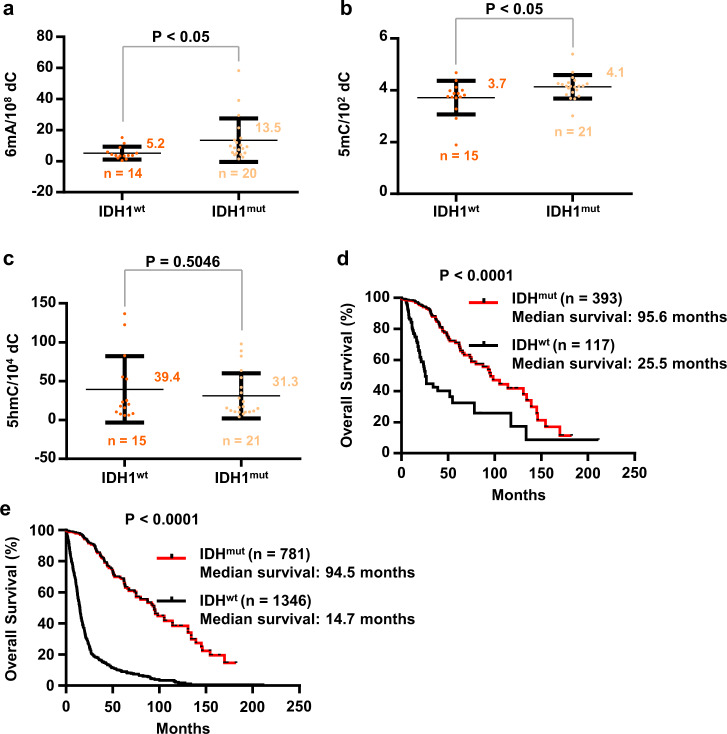
Increase of DNA 6mA and 5 mC in IDH1-mutant gliomas (glioblastoma excluded). **a** DNA 6mA content in IDH1^wt^ and IDH1^mut^ glioma tissues. IDH1^wt^, *n* = 14; IDH1^mut^, *n* = 20. **b**, **c** DNA 5mC (**b**) or 5hmC (**c**) content in IDH1^wt^ and IDH1^mut^ glioma tissues. IDH1^wt^, *n* = 15; IDH1^mut^, *n* = 21. **c** Kaplan–Meier curve of Low-Grade glioma patients’ survival. Patients were stratified into the “IDH1^mut^” group (*n* = 393) and the “IDH1^wt^” group (*n* = 117). **e** Kaplan–Meier curve of Low-Grade glioma and glioblastoma patients’ survival. Patients were stratified into the “IDH1^mut^” group (*n* = 781) and the “IDH1^wt^” group (*n* = 1346). **a**–**c** The glioblastomas were excluded. Statistical significance was determined by Student’s unpaired *t*-test. **d**, **e** Data were obtained from The Cancer Genome Atlas clinical dataset. Significance was determined by log-rank analysis.

Isocitrate dehydrogenase 1 (*IDH1*) and isocitrate dehydrogenase 2 (*IDH2*) are commonly mutated in most low-grade gliomas and secondary glioblastoma multiforme^26,27^, and IDH mutation status is a feature of glioma subclassifications in the 2016 World Health Organization classification^28^. The production of D-2-hydroxyglutarate (2-HG) by mutated *IDH1/2* may inhibit demethylation via competitively displacing the cofactor α-ketoglutarate of known demethylases^29,30^. By similar mechanism, it is also possible to inhibit the elimination of the misincorporated DNA 6mA. We subsequently inquired the correlation between IDH1 mutation and DNA 6mA (Fig. 4). Since only one *IDH1*-mutated patient was found in GBM patients, GBM patients were excluded in this statistical calculation. Compared with *IDH1*^wt^ glioma tissues, DNA 6mA contents were significantly increased in *IDH1*^mut^ glioma tissues (Fig. 4a) (*P* < 0.05). The average 6mA levels of glioma tissues of wt and mut (excluding GBM) are about 5.2 and 13.5 per 10^8^ dC, respectively. Similarly, we found a significant difference of 5mC contents between *IDH1*^wt^ and *IDH1*^mut^ glioma tissues (Fig. 4b) (*P* < 0.05). The average 5mC levels of glioma tissues of wt and mut (excluding GBM) are about 3.7 and 4.1 per 100 dC, respectively. However, no significant difference was observed for 5hmC (Fig. 4c). Consistent with previous literature^26^, *IDH* mutation increases the overall survival (Fig. 4d, e).

### Demethylase candidate ALKBH1 and the erasing of misincorporated DNA 6mA

ALKBH1 has been proposed as a candidate demethylase of DNA 6mA in a number of reports^10,12,13,31^. There is a possibility that the methylated dA is erased immediately by the potential demethylase ALKBH1. Therefore, we knocked down ALKBH1 using siRNAs in four glioblastoma cell lines accompanying with [^15^N_5_]-dA treatment. The depletion of ALKBH1 was confirmed by RT-PCR (Supplementary Fig. S7a). As measured by UHPLC-MS/MS assay, we did not observe consistent increase in DNA 6mA (Supplementary Fig. S7b). Previous report suggested a correlation between ALKBH1 mRNA expression and the overall survival of GBM^13^. Therefore, by employing the online database Gene Expression Profiling Interactive Analysis (GEPIA)^32^, Kaplan–Meier and log-rank analyses were also performed to re-evaluate the differences in survival rates. However, high expression of ALKBH1 mRNA did not alter overall survival (OS) rates of both GBM (Supplementary Fig. S8a) and low-grade glioma (LGG) (Supplementary Fig. S8b). Regarding that the glioblastoma DNA 6mA is the misincorporated product, it is reasonable that potential demethylase ALKBH1 expression is not associated with OS rates of GBMs.

### The level of misincorporated DNA 6mA is correlated with overall survival in human glioblastoma

**Fig. 5:**
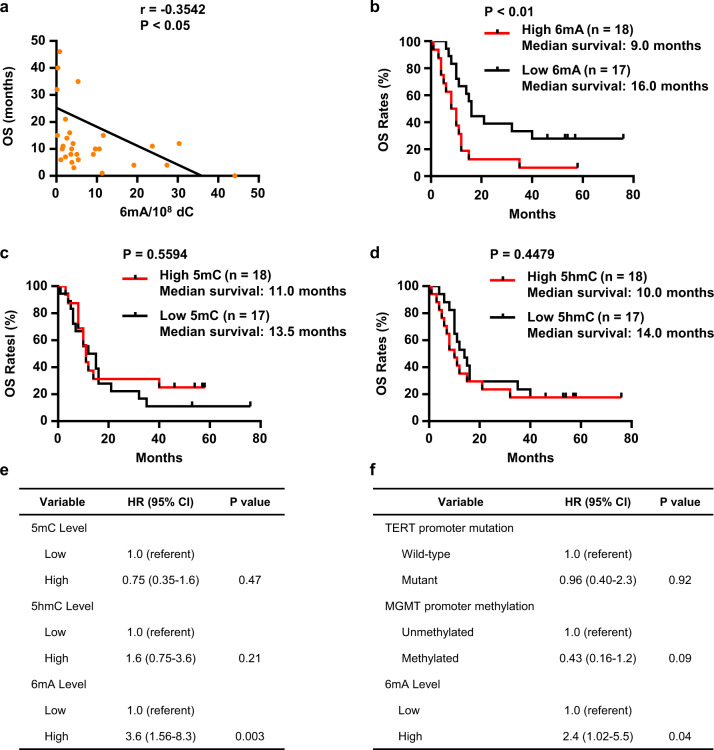
The level of misincorporated DNA 6mA is correlated with overall survival rates in human glioblastomas. **a** Pearson correlations between 6mA level and overall survival time of GBM patients (*n* = 35). **b** Kaplan–Meier curve of GBM patients survival according to 6mA levels in GBM tissues. Patients were stratified into the “high level 6mA” group (*n* = 18) and the “low level 6mA” group (*n* = 17). **c**, **d** Kaplan–Meier curve of GBM patients survival according to 5mC (**c**) or 5hmC (**d**) levels in GBM tissues. Patients were stratified into the “high level 5mC/5hmC” group (*n* = 18) and the “low level 5mC/5hmC” group (*n* = 17). **e** Multivariate Cox proportional hazards ratios for survival based on 6mA level adjusted for 5mC and 5hmC of patients. **f** Multivariate Cox proportional hazards ratios for survival based on 6mA level adjusted for TERT promoter mutation and MGMT promoter methylation of patients. **a** Statistical significance was determined by Student’s unpaired *t*-test. **b**–**d** Significance was determined by log-rank analysis.

We further investigated the impacts of the misincorporated DNA 6mA in patients’ survival. We found that, despite of its rare abundance, the DNA 6mA content in genomic DNA was astonishingly associated with prognosis. By Pearson correlation analysis, it was found that the OS rate of the GBM patients is reversely proportional to the content of DNA 6mA (*P* < 0.05) (Fig. 5a). Consistently, Kaplan–Meier analysis of GBM patients showed that the patients with low DNA 6mA content in GBM tissues have better OS rates than those with high 6mA content (*P* < 0.01) (Fig. 5b). Specifically, the median OS rates for patients with low and high 6mA content are 16 months and 9.0 months, respectively. In contrast, the contents of 5mC and 5hmC at the global levels are not associated with the prognosis. Both the contents of DNA 5mC (*P* = 0.5307) and 5hmC (*P* = 0.4769) are not proportional to the overall survival (Supplementary Fig. S9a, b), and no significant difference in OS was found between patients with the low and high DNA 5mC (*P* = 0.5594) and 5hmC (*P* = 0. 4479) contents (Fig. 5c, d).

Of note, we did not find any link of DNA 6mA with any of the gender, age, promoter methylation status of *MGMT* (O-6-methylguanine DNA methyltransferases), and *TERT* (Telomerase Reverse Transcriptase) mutation of the patients (Supplementary Fig. S10). By the use of Cox Proportional-Hazards Model for multivariate analysis^33^, we adjusted age and gender, finding that 6mA is still significant for correlating with OS rate (Supplementary Table S3a). By simultaneously adjusting age, *MGMT* promoter methylation status and *TERT* mutation, 6mA is also significant for correlating with OS rate (Supplementary Table S3b). By adjusting the levels of 5mC and 5hmC, 6mA is significant for correlating with OS rate (Fig. 5e). By adjusting *MGMT* promoter methylation status and *TERT* mutation, 6mA is significant for correlating with OS rate (Fig. 5f). In contrast, by adjusting age and gender, both 5mC and 5hmC are not significant for the correlation of OS rate (Supplementary Table S4)

Collectively, our data strongly support the link of DNA 6mA contents with patients’ OS rate in human glioblastoma.

### Release of RNA m^6^A nucleoside by cytotoxic stresses and the generation of misincorporated DNA 6mA

**Fig. 6:**
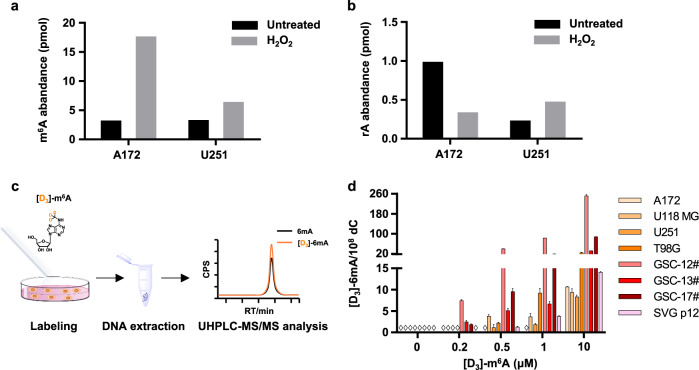
Cytotoxic stimuli facilitates the release of degraded RNA m^6^A nucleoside to extracellular fluid and induces the misincorporated DNA 6 mA in neibourghing cells. **a**, **b** The release of m^6^A (**a**) or rA (**b**) nucleoside from glioblastoma cells after H_2_O_2_ treatment. Glioblastoma cells were treated for 24 h with or without 1 mM H_2_O_2_, and then supernatants were collected and subjected to LC-MS/MS analysis. **c** Flow diagram of stable isotope labeling for tracing genomic incorporation. **d** Quantification of genomic DNA [D_3_]-6mA in in glioma cell lines, GSCs, and glial cell line. The cells were treated with [D_3_]-m^6^A at the concentration as indicated. (◊ indicated ND (not detected)).

As shown recently, extracellular m^6^A is excreted as metabolic end products of RNA breakdown and enhanced by external stimuli in HEK293 cells^34^. Currently, no related data are available for gliomas. Following this hint, we treated glioblastoma cells with cytotoxic reagents. The extracellular nucleosides of m^6^A and rA were extracted and measured by UHPLC-MS/MS. Indeed, hydrogen peroxide (H_2_O_2_) (Fig. 6a) and lipopolysaccharide (LPS) (data not shown) increased extracellular m^6^A. Notably, when cells were treated with cytotoxic reagents, the extracellular levels of unmodified adenosines (rA) did not increase but decreased (Fig. 6b). Of note, hypoxia reduced the amount of extracellular m^6^A nucleoside upon cytotoxic stimuli (data not shown). This is also consistent with a recent work^34^.

**Fig. 7:**
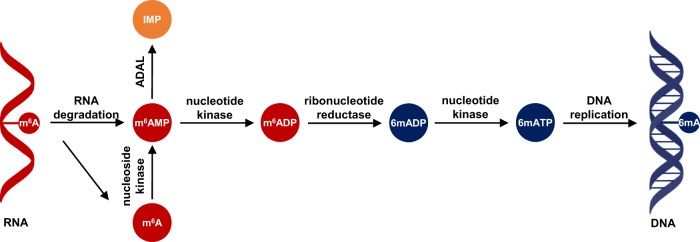
The proposed pathway for the misincorporation of DNA 6mA. Misincorporated DNA 6mA is associated with RNA m^6^A decay and purine salvage synthesis pathway.

Since the observed DNA 6mA is generated through defective DNA replication^20,21^, we attributed the misincorporation of DNA 6mA to be associated with the purine salvage of m^6^A nucleoside (Fig. 7). Following this inference, we further assessed the misincorporation of DNA 6mA in glioma via extracellular m^6^A nucleoside. For this purpose, we treated four glioblastoma cell lines and three GSCs with the modified nucleoside [D_3_]-m^6^A (Fig. 6c). As expected, for all the tested cells, we did observe the misincorporation of DNA [D_3_]-6mA in a dose-dependent manner (Fig. 6d), confirming the reasonability of the observed 6mA resulting from the misincorporation. Noteworthily, extracellular m^6^A can be incorporated the genomes of GSC-12# and GSC-13# as low as the [D_3_]-m^6^A concentration was 0.2 μM.

### The m^6^A nucleoside selectively preserves a subset of the cells and stimulates their stemness and proliferation

**Fig. 8:**
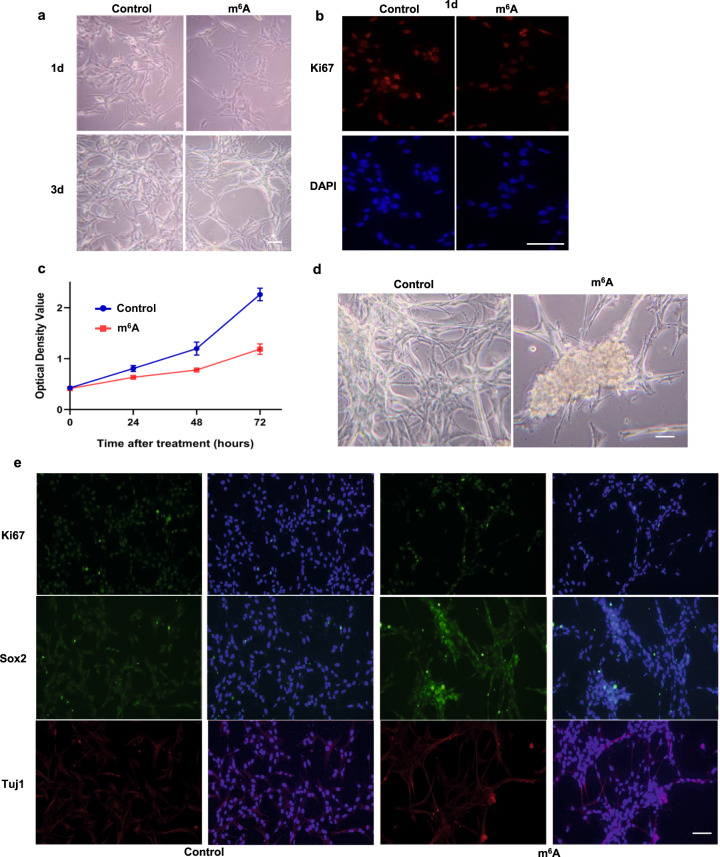
m^6^A treatment selectively preserves a subset of the cells and stimulates their stemness and proliferation. **a**–**c** Progressive inhibition of U87 cell proliferation by m^6^A treatment, as shown by phase light images (**a**), Ki67 staining (**b**), and CCK8 assay (**c**). Scale bar: 50 μm. **d**, **e** Selective preservation of a subset of GBM cells with potential stem cell-like properties. Formation of neurosphere-like structures with m^6^A treatment (**d**); Strong Ki67 (**e**, upper panel) and Sox2 staining (**e**, middle panel) with the neurosphere-like objects; Enhanced Tuj1 staining at the peripherals of the neurosphere-like objects (**e**, lower panel). Scale bar: 50 μm.

We subjected GBM cells to the treatment of m6A nucleoside and observed a strong inhibition of the cell proliferation (Fig. 8a–c). A decrease in proliferation (indicated by Ki67^+^ cells) was already evident with a 24-h treatment (Fig. 8b) and overtime the inhibition was progressive as determined by CCK8 activity (Fig. 8c). Despite a strong inhibition in U87 cell proliferation, intriguingly we have also noticed that there was an increase in neurosphere-like objects with the m^6^A nucleoside treatment group (Fig. 8d). Further staining confirmed that the majority of the cells in the neurosphere-like objects were proliferating with even higher level of Ki67, a proliferation marker, and Sox2, a stem cell marker (Fig. 8e). The cells in the peripheral stained stronger for Tuj1, neuronal marker (Fig. 8e). Taken together, we suspected that m^6^A nucleoside treatment might selectively preserve a subset of the cells and stimulate their stemness and proliferation.

## Discussion

### Non-epigenetic and DNA polymerase-dependent misincorporation of DNA 6mA

Herein, we demonstrated that the observed DNA 6mA is extremely rare with an abundance of ~0.02–0.6 ppm, which is three to four orders of magnitude lower compared to the previous report (~1000 ppm)^13^. By utilizing two heavy stable isotope-labeling strategies, we could trace methylase-generated genomic 6mA in four glioblastoma cells and three glioblastoma stem cells (or primary glioblastoma cells). Surprisingly, we did not detect any labeled-6mA, which indicated that the origin of 6mA in glioma is independent of DNA methyltransferase. On the other hand, these observations support the absence of 6mA methyltransferases-dependent DNA 6mA in human glioblastoma and glioblastoma stem cells.

### Reduced DNA 6mA in human glioma and hypoxia

Although the misincorporated DNA 6mA has been found in a number of culturing human cells^20,21^, the roles of the misincorporated 6mA in diseases are not explored yet. Since it is the most common type of primary brain tumors and accounts for 81% of primary malignant intracranial tumors in human^35^, glioma is of intensive interest. Among three types of human gliomas, low-graded astrocytoma (A), anaplastic astrocytoma (AA), and glioblastoma (GBM)^28,35^, GBM is the most lethal one^36–38^. The median survival of patients with GBM remains < 16 months after diagnosis^39^. So far, no treatment regimen has been found that can significantly prolong the overall survival of GBM patients^39,40^.

Interestingly, we found that compared with normal brain tissues, the abundance of the misincorporated DNA 6mA significantly decreases in all tested human glioma subtypes, including human glioblastomas. We also detected DNA 5mC in glioma from the same set of patients; and by excluding *IDH1* mutation, we observed a significant reduction in human Anaplastic Astrocytomas and glioblastomas, but not in Astrocytoma tissues. Evidently, only can the alteration in the frequency of the non-epigenetic and misincorporated DNA 6mA discriminate all types of human glioma (Astrocytoma, Anaplastic Astrocytoma, and Glioblastoma) from normal brain tissues regardless of *IDH1* mutation.

By culturing glioblastoma cells under hypoxia conditions (1% O_2_), the observed DNA 6 mA greatly reduced. Therefore, the reduction in misincorporated DNA 6mA may be related to hypoxia milieu often suffered in human gliomas.

### The DNA 6mA content is a potential biomarker of overall survival rate in human glioblastoma

Benefiting from bacterial DNA contamination-free and ultrasensitive UHPLC-MS/MS assay, we accurately measured extremely rare DNA 6mA in human glioblastomas. We investigated the statistic link of DNA 6mA with any of the age, gender, promoter methylation status of MGMT, TERT mutation, and *IDH1/2* mutations of the patients (Supplementary Fig. S10), and we did not find any link for these factors except *IDH* mutation. Therefore, our data suggest that these factors cannot affect the level of DNA 6mA except *IDH* mutation. Both Pearson correlation analysis and Kaplan-Meier analysis consistently support the correlation of DNA 6mA with the OS rates of GBM patients. The median OS rates for patients with low 6mA content is 1.78-fold longer than that for patients with high 6mA content. By the use of Cox Proportional-Hazards Model^33^, the most commonly used multivariate approach, we did not find any of age, gender, *MGMT* promoter methylation status, and *TERT* mutation affecting the correlation of 6mA with OS rate.

Although its depletion was found in many types of human cancer^41^, the reported link of 5hmC with poor survival of glioma patients is not consistent^42–44^. Intriguingly, our data suggest that both the 5mC and 5hmC contents at the global levels are not associated with the prognosis. By the use of Cox Proportional-Hazards Model, 5mC and 5hmC do not alter the correlation of 6mA with the OS rates. By same approach considering age and gender, both 5mC and 5hmC are not significant for the correlation of OS rate. Pearson correlation analysis does not support the correlation of 5hmC with OS rates, too. All these data strongly suggest that among the three modified deoxynucleosides of genomic DNA (6mA, 5mC, and 5hmC), 6mA is probably the best biomarker for predicting prognosis.

### Implications on the increase of the misincorporated DNA 6mA by IDH1 mutation

Multivariate analysis confirmed that *IDH1* mutation (R132H) was an independent favorable prognostic marker in gliomas^45^. Importantly, *IDH1* mutation confers a distinctive survival advantage in glioma patients; large cohort studies confirmed a 2-fold increase of median overall survival in glioblastoma patients and a more than threefold increase in lower-grade glioma patients compared with their respective controls^46,47^. Our data also support the conferred survival advantage of *IDH1* mutation.

In addition, we also observed that *IDH1* mutation increases the misincorporated DNA 6mA in human gliomas, hinting that the misincorporated DNA 6mA can be erased in vivo. It is also possible that the post-replicative and epigenetic 6mA was erased by the proposed demethylase candidate ALKBH1 in glioma. However, we neither observed any labeled-6mA nor an explicit change of 6mA contents upon the depletion of AKLBH1. Therefore, non-epigenetic and misincorporated DNA 6mA is solely present and generated in a methyltransferase-independent and DNA polymerase-mediated misincorporation. Taken together, our data strongly suggest that there might be an unknown demethylase responsible for erasing the misincorporated DNA 6mA.

### The misincorporated DNA 6mA in human glioblastoma reflects cytotoxic stresses

The observed DNA 6mA originates from the misincorporating action of DNA polymerase during DNA replication or repair-related amplification^20,21^. Such misincorporation should be a reflection of the purine salvage synthesis of m^6^A nucleoside, e.g., extracellular m^6^A nucleoside. Interestingly, extracellular m^6^A is excreted as stimulated by external toxic reagents in HEK293 cells^34^. By treating glioblastoma cells with cytotoxic reagents (H_2_O_2_ and LPS), we also observed the release of the nucleosides of m^6^A and rA to extracellular medium, which were extracted and measured by UHPLC-MS/MS. Despite of abundant rA in cellular RNA, the amount of released rA nucleoside is 10-fold lower than that of m^6^A nucleoside. Essentially, the m^6^A nucleoside released from stressed cells can be re-used by stress-free neighboring cells via uptake followed by purine salvage synthesis and DNA polymerase-dependent misincorporation. Therefore, the misincorporated DNA 6mA is a measure of stress-induced release of m^6^A nucleoside from RNA breakdown. In a simple word, the content of misincorporated DNA 6mA reflects the cellular stresses.

In summary, we demonstrate extremely low DNA N^6^-methyladenine in human glioma (including glioblastoma) and attributed the observed DNA 6mA to the DNA polymerase-dependent misincorporation. The misincorporated DNA 6mA significantly decreases in human glioma compared to normal brain tissues. Noteworthily, DNA 6mA content is correlated with overall survival rates. Essentially, our findings may open up avenues for targeting early diagnosis and prognosis for the glioma patients.

## Materials and methods

### Chemicals and materials

2′-deoxycytidine (dC, ≥99%), 2′-deoxyadenosine monohydrate (dA·H_2_O, ≥ 99%), adenosine (rA, ≥ 99%), and formic acid (for mass spectrometry, ≈ 98%) were purchased from Sigma-Aldrich (St. Louis, MO, USA). 5-methyl-2′-deoxycytidine (5mC) were ordered from Berry & Associates (Dexter, MI). [^15^N_5_]-2′-deoxyadenosine ([^15^N_5_]-dA), [^15^N_3_]-2′-deoxycytidine ([^15^N_3_]-dC), [D_3_]-5-methyl-2′-deoxycytidine ([D_3_]-5mC), N^6^-methyladenosine (m^6^A), and [D_3_]-L-methionine were obtained from Cambridge Isotope Laboratories (Andover, MA, USA). N^6^-methyl-2′-deoxyadenosine (6mA) was purchased from Santa Cruz Biotechnology, Inc. (Dallas, Texas, USA). [D_3_]-N^6^-methyladenosine ([D_3_]-m^6^A) was ordered from Toronto Research Chemicals (North York, Ontario, Canada). [^15^N_5_]-N^6^-methyl-2′-deoxyadenosine ([^15^N_5_]-6mA) was synthesized and purified in our own lab. Ammonium bicarbonate (NH_4_HCO_3_) was ordered from Merck KGaA (Darmstadt, Germany). Ultrapure water was produced using ELGA PureLab-water purification system (High Wycombe, Bucks, UK). Crotalus adamanteus venom phosphodiesterase I (SVP) was obtained from Worthington Biochemical Corporation (Lakewood, CO, USA). Deoxyribonuclease I (DNase I) and calf intestinal alkaline phosphatase (CIP) was purchased from New England Biolabs (Ipswich, MA, USA). Other chemicals of at least analytical reagents were used.

### Patients and samples

All the glioma samples were obtained with informed consent under a protocol approved by the Institutional Review Board of Peking Union Medical College Hospital. All the tissues were obtained at the time of surgery (before any radiotherapy or chemotherapy) and cryopreserved at –80 °C until use. Under the approval by the institutional review board of the institute of Basic Medical Science, Chinese Academy of Medical Science (Approval Number: 009–2014), all normal brain tissues without signs of neurological disorders were provided from the National Human Brain Bank for Development and Function, Chinese Academy of Medical Sciences & Peking Union Medical College. The clinicopathological characteristics of patients are shown in Table 1.

This study was compliant with all relevant ethical regulations of Institute of Basic Medical Sciences, Chinese Academy of Medical Sciences regarding research involving human participants (Approval Number: 004–2016).

### Cell culture

The human glioblastoma A172, U118 MG, U251, and T98G cell lines were purchased from National Infrastructure of Cell Line Resource (Beijing, China). SVG p12 cell line was purchased from American Type Culture Collection (Manassas, VA, USA). A172, U118MG, and U251 cell lines were cultured in DMEM high glucose medium (Thermo Fisher Scientific, Waltham, MA, USA) with 10% fetal bovine serum (Gibco, Grand Island, NY, USA). T98G cell line was cultured in MEM/EBSS medium (HyClone Laboratories, Logan, UT, USA) with 10% fetal bovine serum (Gibco, Grand Island, NY, USA). SVG p12 cell line was cultured in EMEM medium (ATCC, Manassas, VA, USA) with 10% fetal bovine serum (Gibco, Grand Island, NY, USA). The GSC-12#, GSC-13#, GSC-17# were obtained from fresh surgical specimens of human GBMs under the approval by the Tongji Hospital, Tongji University School of Medicine and the consent with the patients’ agreements. The GSCs were cultured in DMEM/F12 medium (Gibco, Grand Island, NY, USA) supplemented with 1× GlutaMAX (Thermo Fisher Scientific, Waltham, MA, USA), 1× B-27 supplement (Thermo Fisher Scientific, Waltham, MA, USA), 1× N-2 Supplement (Thermo Fisher Scientific, Waltham, MA, USA), 20 ng/mL epidermal growth factor (EGF) (PeproTech, Rocky Hill, NJ, USA), 20 ng/mL fibroblast growth factor (FGF) (Proteintech Group, Rosemont, IL, USA), and 10 μg/mL heparin (Sigma-Aldrich, St. Louis, MO, USA). Only early-passage cells were used for the study and all cells were cultured in a humidified incubator at 37 °C supplemented with 5% CO_2_.

### The treatment of cultured cells with [^15^N_5_]-dA, [D_3_]-l-methionine, or [D_3_]-m^6^A

In all, 1.0 × 10^5^ cells of glioma cell lines and GSCs were seeded in 6-well cell culture cluster (Corning, Corning, NY, USA), and cultured in 2.0 mL medium. The final concentration of treated [^15^N_5_]-dA was 20 μM, and the final concentration of treated [D_3_]-l-methionine was 30 μg/mL. The final concentration of treated [D_3_]-m^6^A was ranged from 0.2 to 10 μM. The cells were treated with [^15^N_5_]-dA or [D_3_]-L-methionine for 4 days, or with [D_3_]-m^6^A for 2 days and harvested for DNA extraction and UHPLC-MS/MS analysis.

### siRNA transient transfection

Glioma cells were seeded into six-well plates at a concentration of 1 × 10^5^ cells/2 mL per well for 48 h. Then, the cells were transfected with 25 pmol siRNA (GenePharma, Suzhou, China) using Lipofectamine RNAiMAX (Thermo Fisher Scientific, Waltham, MA, USA) following the manufacturer’s protocols and incubated for 48 h. The cells were harvested for DNA extraction and RNA extraction, respectively. The mRNA expression level of ALKBH1 was measured by real-time quantitative PCR (RT-PCR). The siRNA oligonucleotides sequences are listed in Supplementary Table S1. The RT-PCR primer sequences are listed in Supplementary Table S2.

### DNA and RNA extraction

Genomic DNA was extracted using Genomic DNA Purification Kit (Promega, Madison, WI, USA), following the manufacturer’s instructions. For patient tissues extraction, frozen tissues ground using a tissue grinder (TIANGEN, Beijing) were suspended in 600 μL nuclear lysis buffer that contained a final concentration of 20 mM EDTA and 4 U proteinase K (New England Biolabs, Ipswich, MA, USA) and then were incubated overnight at 55 °C with gentle shaking. Subsequent experimental procedures followed the manufacturer’s instructions. The concentration of the extracted DNA was quantified using NanoDrop 2000 (Thermo Fisher Scientific, Waltham, MA, USA) and the DNA quality was evaluated with the ratio of absorbance at 260 nm and 280 nm. Total RNA was extracted from cells using TRIzol reagent (Life technologies Corporation). Messenger RNA was purified from total RNA with two rounds of polyA-tailed purification using Dynabeads^®^ mRNA Purification Kit (Thermofisher Scientific, Waltham, MA, USA).

### Enzymatic digestion of DNA

Genomic DNA (5 μg) was digested into single 2′-deoxyribonucleosides with 1.0 U DNase I, 0.02 U SVP, and 5.0 U CIP at 37 °C overnight as described previously^48^. Finally, DNA samples were filtered by ultra-filtration (MW cutoff: 3 kDa; Pall, Port Washington, NY, USA), and then were subjected to UHPLC-MS/MS analysis.

### UHPLC-MS/MS analysis

The UHPLC-MS/MS analysis was performed on an Agilent 1290 Infinity ultrahigh performance LC system coupled with an ESI-triple quadrupole mass spectrometer (G6410B or G6495, Agilent Technologies, Santa Clara, CA). A Zorbax Eclipse Plus C18 column (2.1 mm × 50 mm, 1.8 μm particle size, Agilent, USA) was employed for the separation of mononucleosides. The column temperature was set at 30 °C.

For dC, dA, rA, and 5mC analysis, the mobile phases consisted of solvent A (water with 0.1% formic acid), and solvent B (pure methanol). The flow rate was 0.3 mL/min, and the injection volume was 2.0 μL. A gradient elution was applied for UHPLC separation: 0–1.5 min, 5.0% B; 1.5−2.0 min, 20.0% B; 2.0−2.3 min, 20% B; 2.3−2.4 min, 5.0% B; 2.4−3.8 min, 5.0% B. For 6mA and m^6^A analysis, the mobile phases consisted of solvent A (water with 2 mM NH_4_HCO_3_), and solvent B (pure methanol). The flow rate was 0.35 mL/min and the injection volume was 10.0 μL. A gradient elution was applied for UHPLC separation: 0–1.0 min, 10.0% B; 1.0−5.0 min, 40.0% B; 5.0−6.0 min, 80% B; 6.0−8.0 min, 80% B; 8.0−8.2 min, 10% B; 8.2−12.0 min, 10% B.

The mass spectrometer was operated under positive ionization mode with 3500 V capillary voltage. A multiple reaction monitoring (MRM) mode was applied: m/z 228 → 112 for dC (5 eV), m/z 252 → 136 for dA (10 eV), m/z 256 → 140 for [^15^N_4_]-dA (10 eV), m/z 257 → 141 for [^15^N_5_]-dA (10 eV), m/z 268 → 136 for rA (10 eV), m/z 242 → 126 for 5mC (5 eV), m/z 245 → 129 for [D3]-5mC (5 eV), m/z 266 → 150 for 6 mA (15 eV), m/z 270 → 154 for [15N4]-6mA (15 eV), m/z 271 → 155 for [^15^N_5_]-6mA (15 eV), m/z 269 → 153 for [D_3_]-6mA (15 eV), and m/z 282 → 150 for m^6^A (15 eV). The fragmentation voltage for all the MRM transitions were set at 90 V to allow efficient transit of precursor ions. Nitrogen gas was used for nebulization and desolvation. The nebulization gas pressure, the source temperature and the flow rate of desolvation gas were respectively set at 40 psi, 300 °C and 9.0 L/min. The collision gas was high purity nitrogen (99.999%).

### Immunofluorescence staining of cells

Immunofluorescence staining was done as previously reported^49^. Briefly After 15-min fixation with 4% (w/v) paraformaldehyde and permeabilization with 0.25% Triton-X-100 in 1× PBS for 15 min, cells were blocked 3% BSA at RT and then incubated with primary antibodies at 4 °C overnight and with secondary antibodies at RT for 2 h. The primary antibodies were rabbit anti-Ki67 (1:1000, Thermo Fisher, MA5–14520), goat anti-Sox2 (1:500, Santa Cruz, sc-17320), rabbit anti-Tuj1 (1:750, Covance, PRB-435P). DAPI was used for counterstaining. Images were acquired with a Nikon ECLIPSE Ti microscope.

### CCK8 assay

CCK8 assays were performed and quantified following the manufacture’ s instruction.

## Supplementary information

Supplementary Materials

## Data Availability

All data from this study are included within this manuscript/Supplementary Material and are available from the Lead Contact (Hailin Wang, hlwang@rcees.ac.cn) upon request.
