# Supplementary material for: Rare and misincorporated DNA N6-methyladenine is a hallmark of cytotoxic stresses for selectively stimulating the stemness and proliferation of glioblastoma cells
Source: Cell Discov. 2022 Apr 30;8:39. doi: 10.1038/s41421-022-00399-x (PMC9061847; doi:10.1038/s41421-022-00399-x)
Supplement: Supplementary file 1 — Supplementary Materials [file 41421_2022_399_MOESM1_ESM.pptx]

## Slide 1
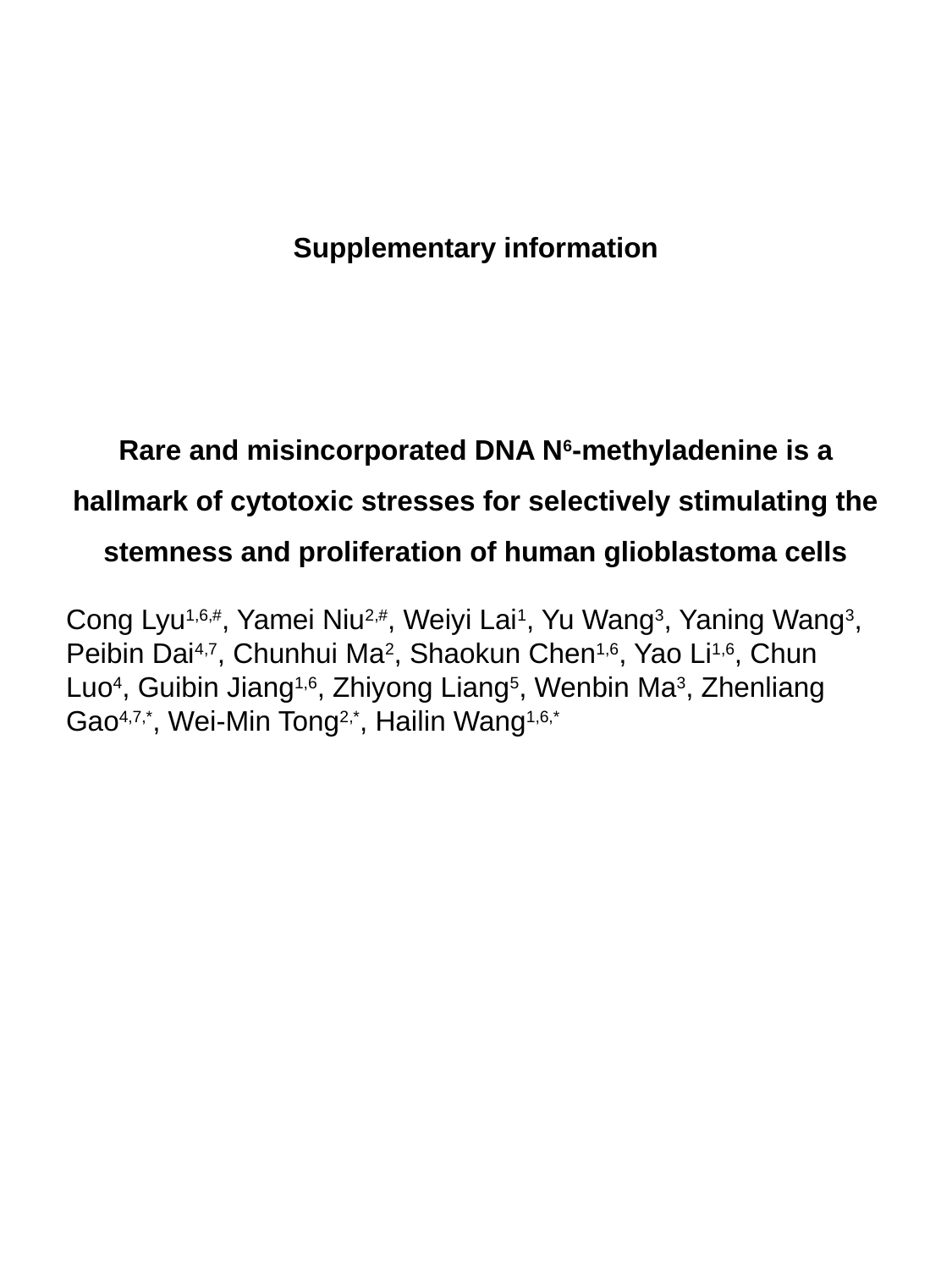

Supplementary information
Rare and misincorporated DNA N6-methyladenine is a hallmark of cytotoxic stresses for selectively stimulating the stemness and proliferation of human glioblastoma cells
Cong Lyu1,6,#, Yamei Niu2,#, Weiyi Lai1, Yu Wang3, Yaning Wang3, Peibin Dai4,7, Chunhui Ma2, Shaokun Chen1,6, Yao Li1,6, Chun Luo4, Guibin Jiang1,6, Zhiyong Liang5, Wenbin Ma3, Zhenliang Gao4,7,*, Wei-Min Tong2,*, Hailin Wang1,6,*

## Slide 2
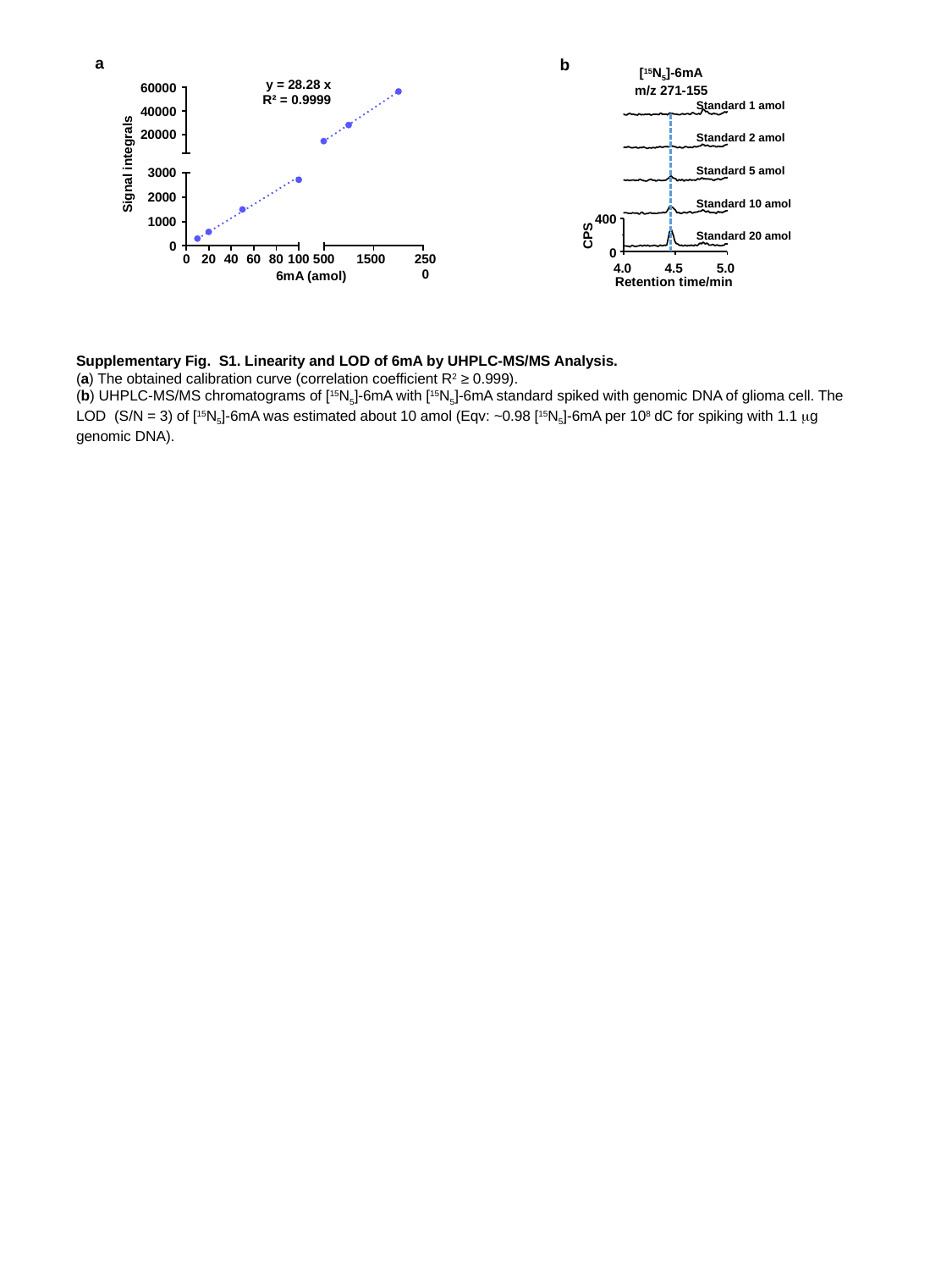

a
b
[15N5]-6mA
m/z 271-155
y = 28.28 x
R² = 0.9999
60000
Standard 1 amol
Standard 2 amol
Standard 5 amol
Standard 10 amol
Standard 20 amol
40000
20000
Signal integrals
3000
2000
400
1000
CPS
0
0
0
20
40
60
80
100
500
1500
2500
4.0
4.5
5.0
6mA (amol)
Retention time/min
Supplementary Fig. S1. Linearity and LOD of 6mA by UHPLC-MS/MS Analysis.
(a) The obtained calibration curve (correlation coefficient R2 ≥ 0.999).
(b) UHPLC-MS/MS chromatograms of [15N5]-6mA with [15N5]-6mA standard spiked with genomic DNA of glioma cell. The LOD (S/N = 3) of [15N5]-6mA was estimated about 10 amol (Eqv: ~0.98 [15N5]-6mA per 108 dC for spiking with 1.1 μg genomic DNA).

## Slide 3
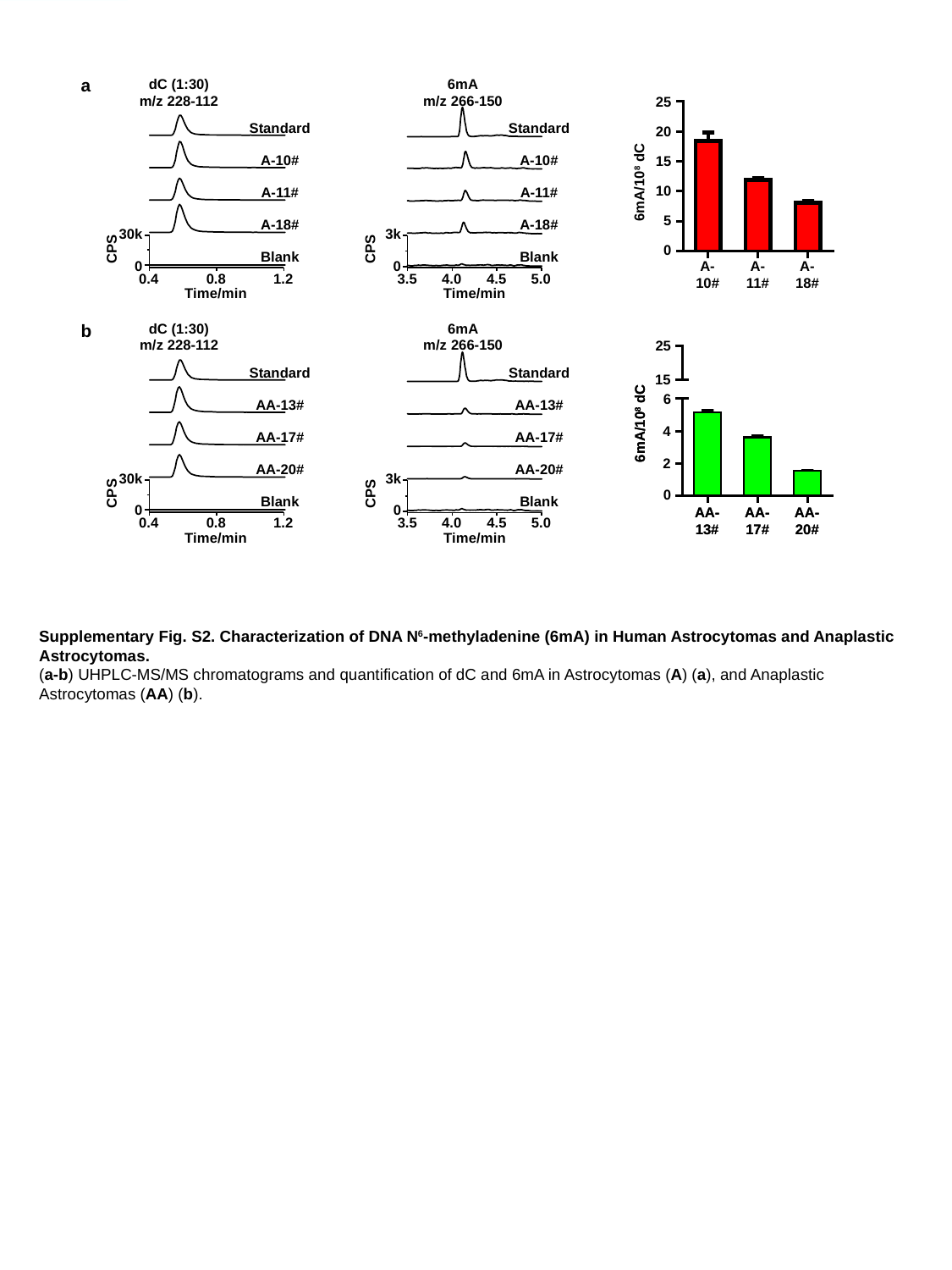

a
dC (1:30)
m/z 228-112
6mA
m/z 266-150
25
20
15
10
5
0
Standard
A-10#
A-11#
A-18#
Blank
30k
CPS
0
0.4
0.8
1.2
Time/min
Standard
A-10#
A-11#
A-18#
Blank
3k
CPS
0
3.5
4.0
4.5
5.0
Time/min
6mA/108 dC
A-10#
A-11#
A-18#
b
dC (1:30)
m/z 228-112
6mA
m/z 266-150
25
15
6
4
2
0
Standard
AA-13#
AA-17#
AA-20#
Blank
30k
CPS
0
0.4
0.8
1.2
Time/min
Standard
AA-13#
AA-17#
AA-20#
Blank
3k
CPS
0
3.5
4.0
4.5
5.0
Time/min
6mA/108 dC
6mA/108 dC
AA-13#
AA-17#
AA-20#
AA-13#
AA-17#
AA-20#
Supplementary Fig. S2. Characterization of DNA N6-methyladenine (6mA) in Human Astrocytomas and Anaplastic Astrocytomas.
(a-b) UHPLC-MS/MS chromatograms and quantification of dC and 6mA in Astrocytomas (A) (a), and Anaplastic Astrocytomas (AA) (b).

## Slide 4
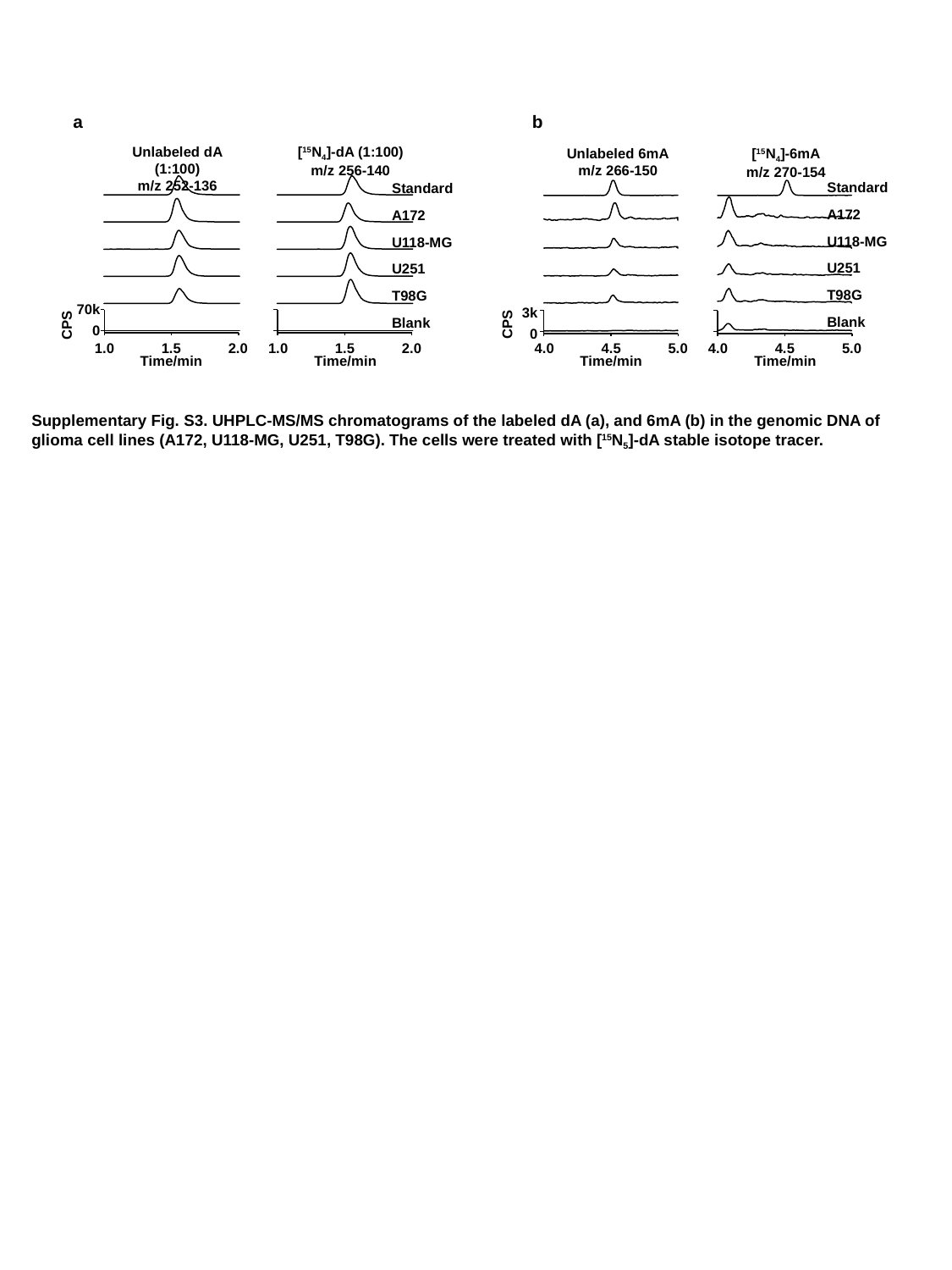

a
b
Unlabeled dA (1:100)
m/z 252-136
[15N4]-dA (1:100)
m/z 256-140
Unlabeled 6mA
m/z 266-150
[15N4]-6mA
m/z 270-154
Standard
A172
U118-MG
U251
T98G
Blank
Standard
A172
U118-MG
U251
T98G
Blank
70k
0
CPS
3k
0
CPS
4.0
4.5
5.0
4.0
4.5
5.0
Time/min
Time/min
1.0
1.5
2.0
1.0
1.5
2.0
Time/min
Time/min
Supplementary Fig. S3. UHPLC-MS/MS chromatograms of the labeled dA (a), and 6mA (b) in the genomic DNA of glioma cell lines (A172, U118-MG, U251, T98G). The cells were treated with [15N5]-dA stable isotope tracer.

## Slide 5
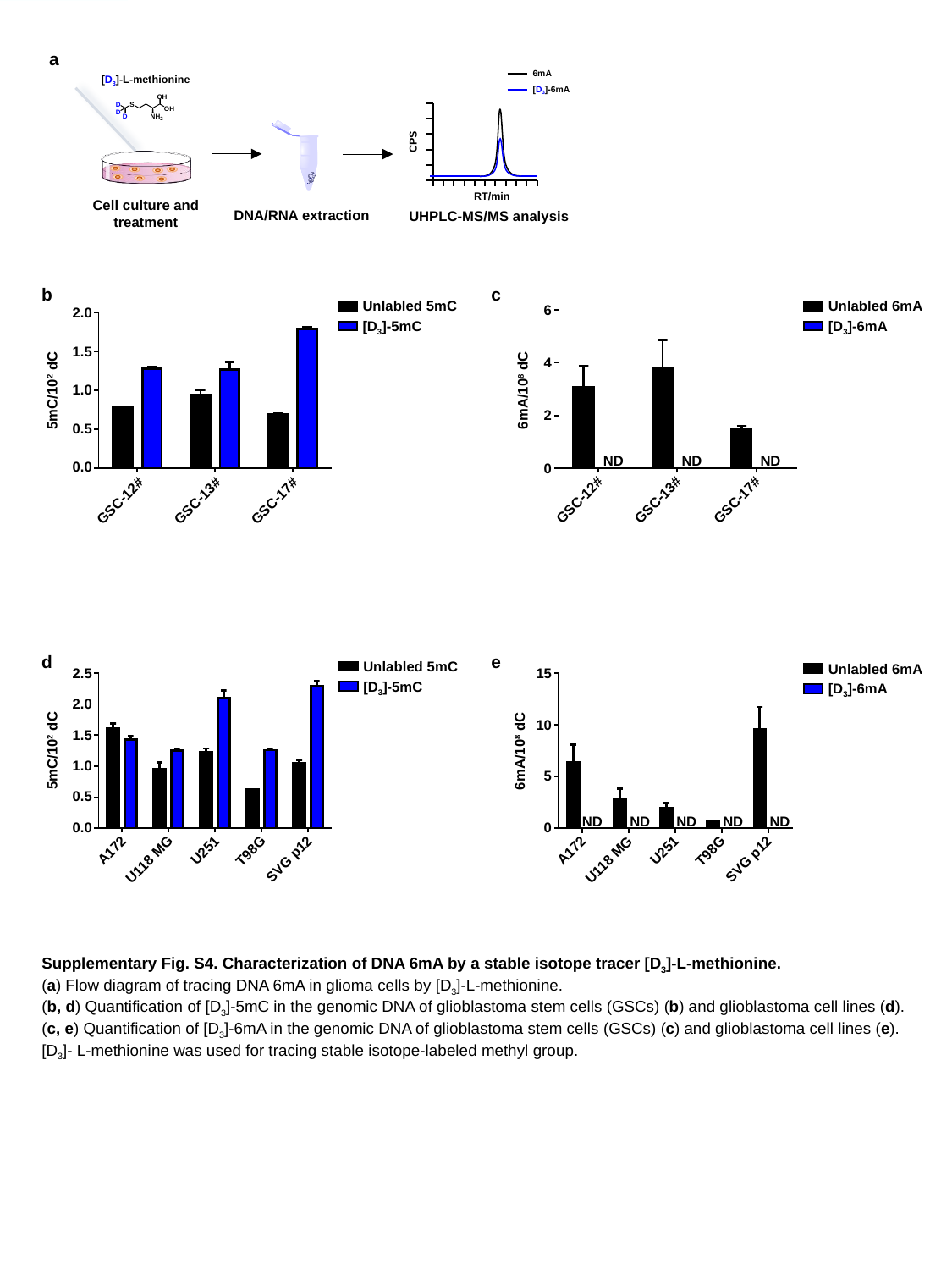

a
6mA
[D3]-L-methionine
[D3]-6mA
### Chart
| Category | |
|---|---|
CPS
RT/min
Cell culture and treatment
DNA/RNA extraction
UHPLC-MS/MS analysis
b
c
Unlabled 5mC
[D3]-5mC
Unlabled 6mA
[D3]-6mA
6
4
2
0
6mA/108 dC
GSC-17#
GSC-12#
GSC-13#
2.0
1.5
1.0
0.5
0.0
5mC/102 dC
GSC-12#
GSC-13#
GSC-17#
ND
ND
ND
d
e
Unlabled 5mC
[D3]-5mC
Unlabled 6mA
[D3]-6mA
2.5
2.0
1.5
1.0
0.5
0.0
5mC/102 dC
T98G
SVG p12
U118 MG
U251
A172
15
10
5
0
6mA/108 dC
T98G
SVG p12
U118 MG
U251
A172
ND
ND
ND
ND
ND
Supplementary Fig. S4. Characterization of DNA 6mA by a stable isotope tracer [D3]-L-methionine.
(a) Flow diagram of tracing DNA 6mA in glioma cells by [D3]-L-methionine.
(b, d) Quantification of [D3]-5mC in the genomic DNA of glioblastoma stem cells (GSCs) (b) and glioblastoma cell lines (d).
(c, e) Quantification of [D3]-6mA in the genomic DNA of glioblastoma stem cells (GSCs) (c) and glioblastoma cell lines (e).
[D3]- L-methionine was used for tracing stable isotope-labeled methyl group.

## Slide 6
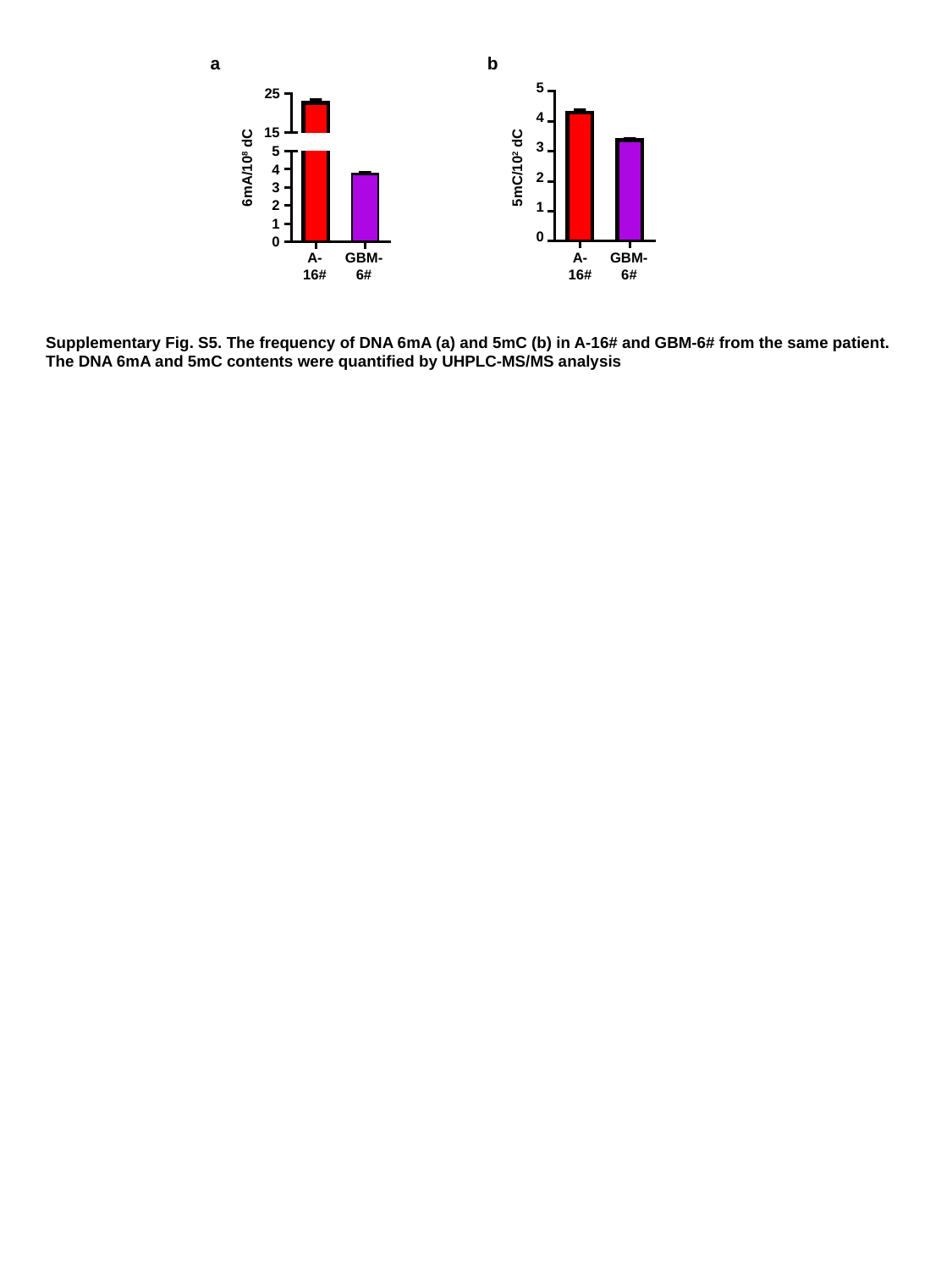

a
b
5
4
3
2
1
0
25
15
5
4
3
2
1
0
5mC/102 dC
6mA/108 dC
A-
16#
GBM-
6#
A-
16#
GBM-
6#
Supplementary Fig. S5. The frequency of DNA 6mA (a) and 5mC (b) in A-16# and GBM-6# from the same patient. The DNA 6mA and 5mC contents were quantified by UHPLC-MS/MS analysis

## Slide 7
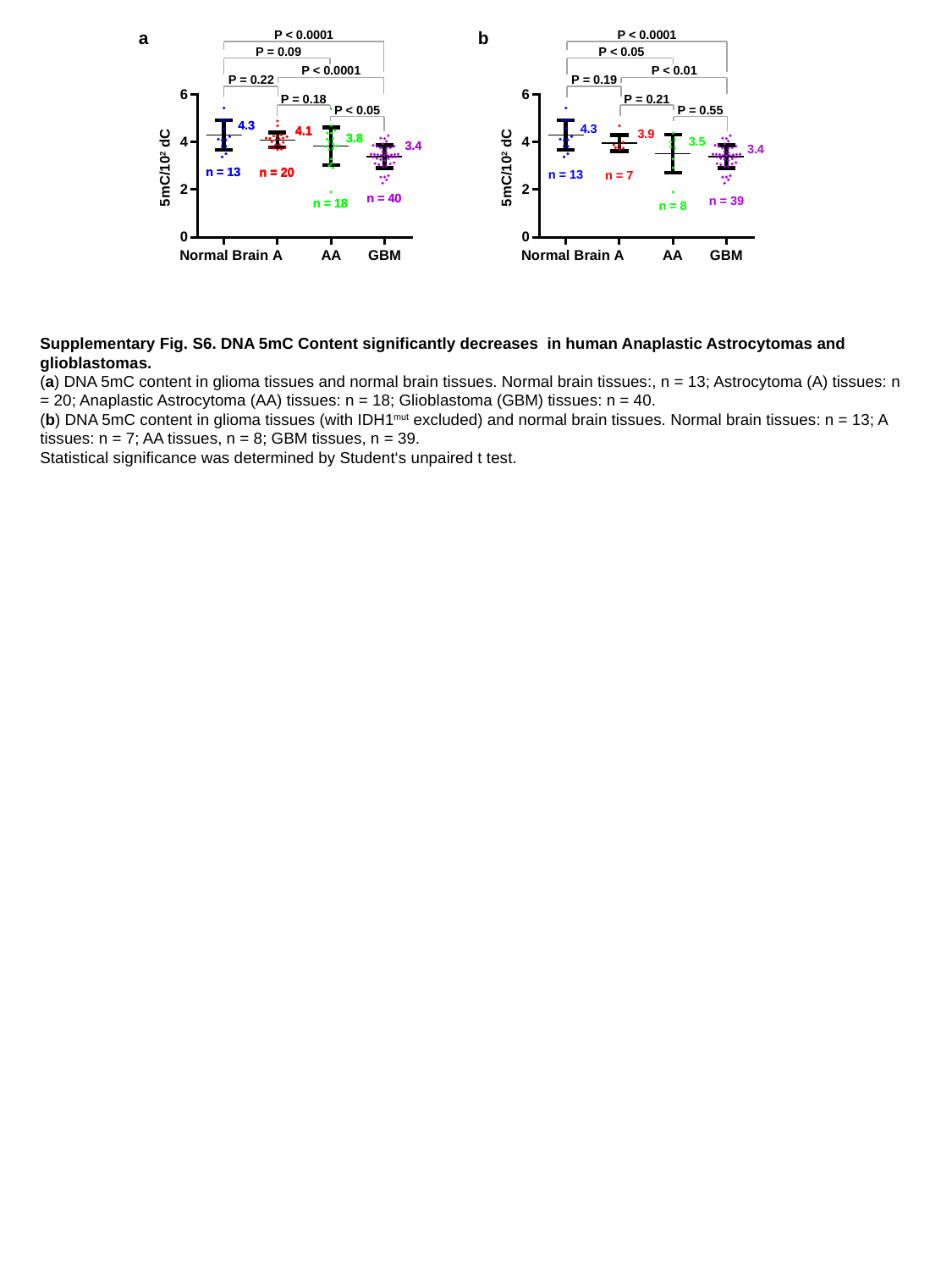

b
a
P < 0.0001
P = 0.09
P < 0.0001
P = 0.22
P = 0.18
P < 0.05
P < 0.0001
P < 0.05
P < 0.01
P = 0.19
P = 0.21
P = 0.55
6
4
2
0
5mC/102 dC
Normal Brain
A
AA
GBM
6
4
2
0
5mC/102 dC
Normal Brain
A
AA
GBM
4.3
4.1
3.8
3.4
4.3
4.1
3.8
3.4
4.3
3.9
3.5
3.4
n = 13
n = 20
n = 40
n = 18
n = 13
n = 20
n = 40
n = 18
n = 13
n = 7
n = 39
n = 8
Supplementary Fig. S6. DNA 5mC Content significantly decreases in human Anaplastic Astrocytomas and glioblastomas.
(a) DNA 5mC content in glioma tissues and normal brain tissues. Normal brain tissues:, n = 13; Astrocytoma (A) tissues: n = 20; Anaplastic Astrocytoma (AA) tissues: n = 18; Glioblastoma (GBM) tissues: n = 40.
(b) DNA 5mC content in glioma tissues (with IDH1mut excluded) and normal brain tissues. Normal brain tissues: n = 13; A tissues: n = 7; AA tissues, n = 8; GBM tissues, n = 39.
Statistical significance was determined by Student‘s unpaired t test.

## Slide 8
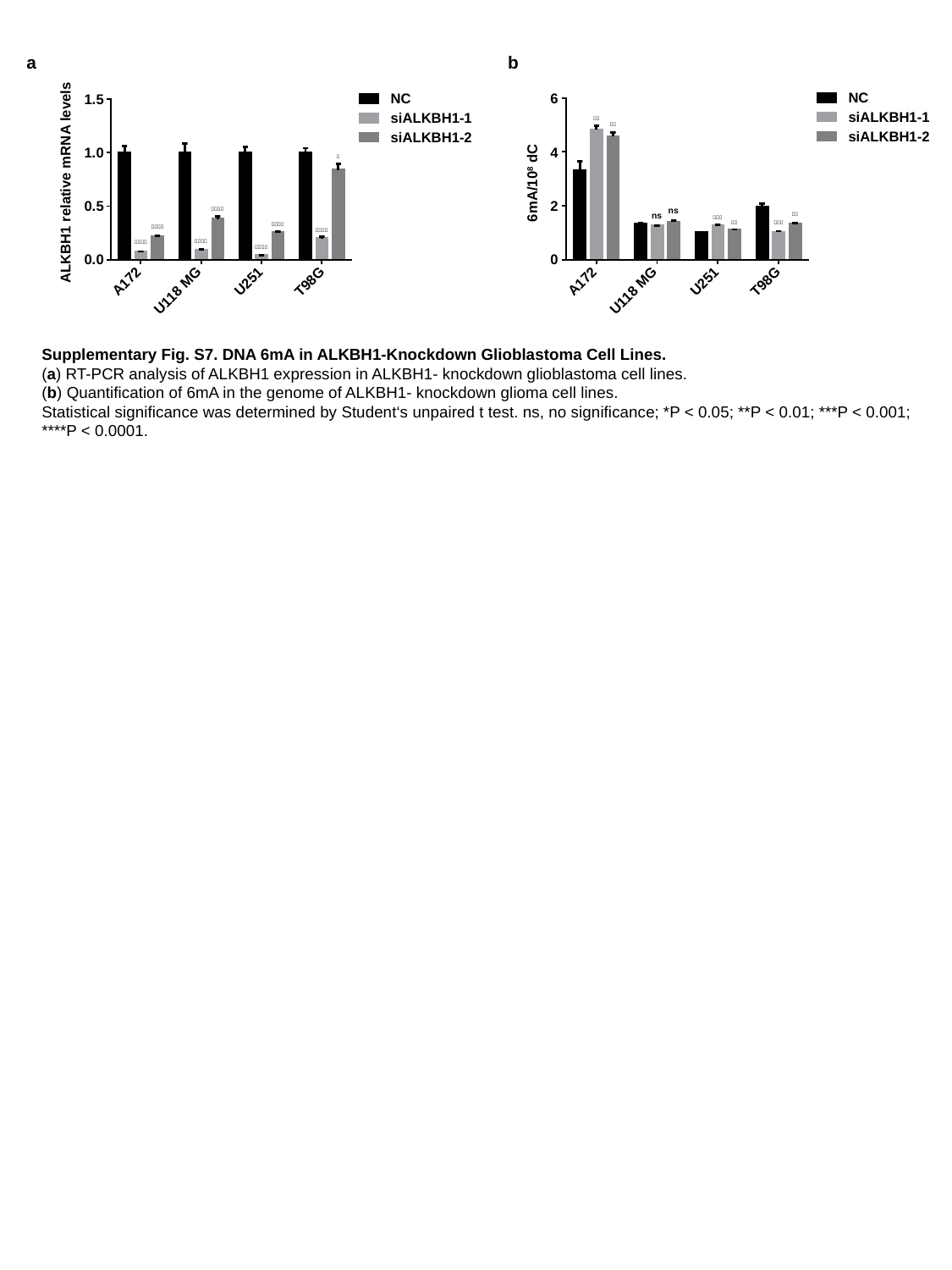

a
b
6
4
2
0
NC
siALKBH1-1
siALKBH1-2
1.5
1.0
0.5
0.0
NC
siALKBH1-1
siALKBH1-2




ALKBH1 relative mRNA levels
6mA/108 dC
ns
ns










T98G
A172
U251
U118 MG
T98G
A172
U251
U118 MG
Supplementary Fig. S7. DNA 6mA in ALKBH1-Knockdown Glioblastoma Cell Lines.
(a) RT-PCR analysis of ALKBH1 expression in ALKBH1- knockdown glioblastoma cell lines.
(b) Quantification of 6mA in the genome of ALKBH1- knockdown glioma cell lines.
Statistical significance was determined by Student‘s unpaired t test. ns, no significance; *P < 0.05; **P < 0.01; ***P < 0.001; ****P < 0.0001.

## Slide 9
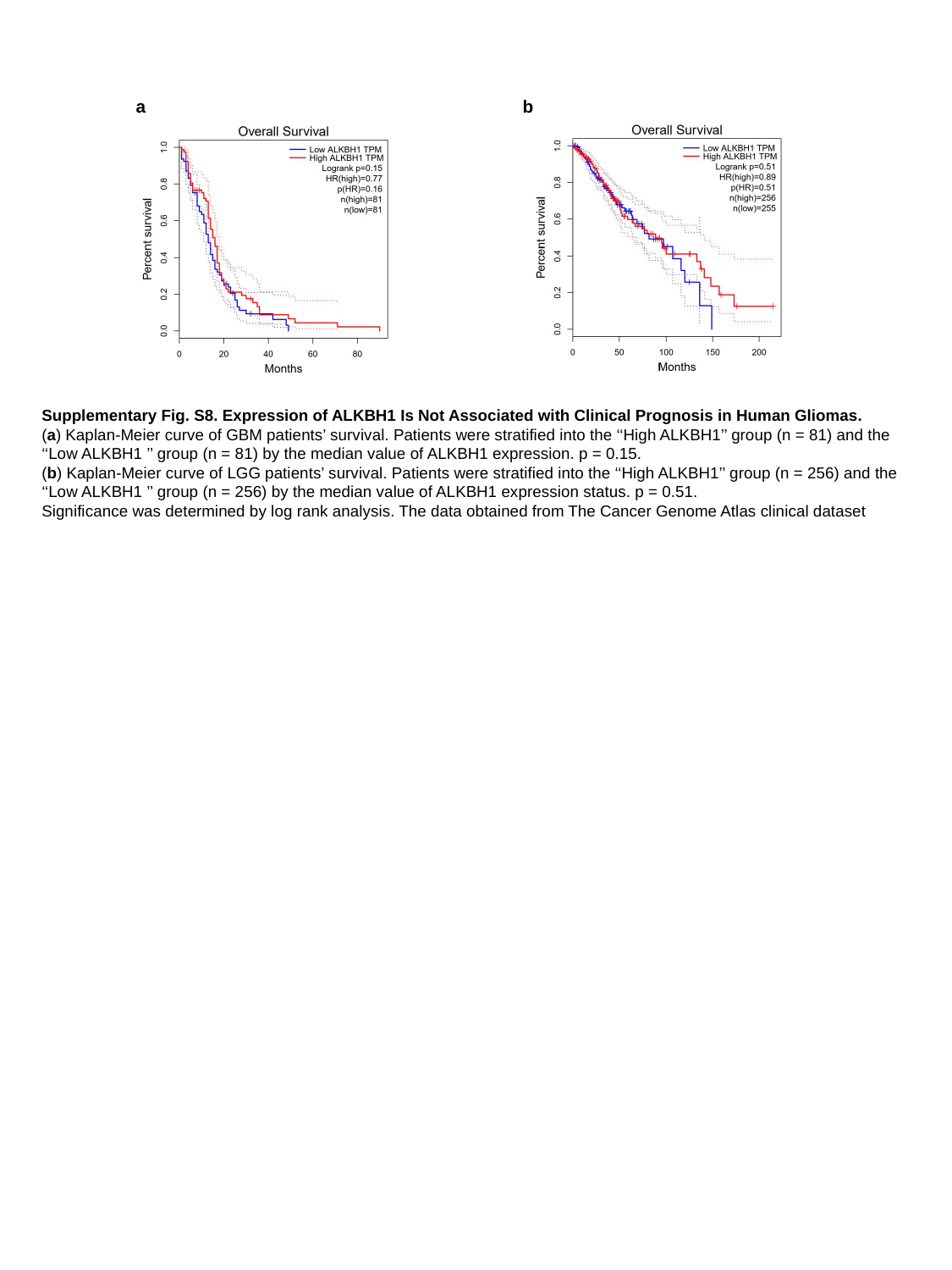

a
b
Supplementary Fig. S8. Expression of ALKBH1 Is Not Associated with Clinical Prognosis in Human Gliomas.
(a) Kaplan-Meier curve of GBM patients’ survival. Patients were stratified into the ‘‘High ALKBH1’’ group (n = 81) and the ‘‘Low ALKBH1 ’’ group (n = 81) by the median value of ALKBH1 expression. p = 0.15.
(b) Kaplan-Meier curve of LGG patients’ survival. Patients were stratified into the ‘‘High ALKBH1’’ group (n = 256) and the ‘‘Low ALKBH1 ’’ group (n = 256) by the median value of ALKBH1 expression status. p = 0.51.
Significance was determined by log rank analysis. The data obtained from The Cancer Genome Atlas clinical dataset

## Slide 10
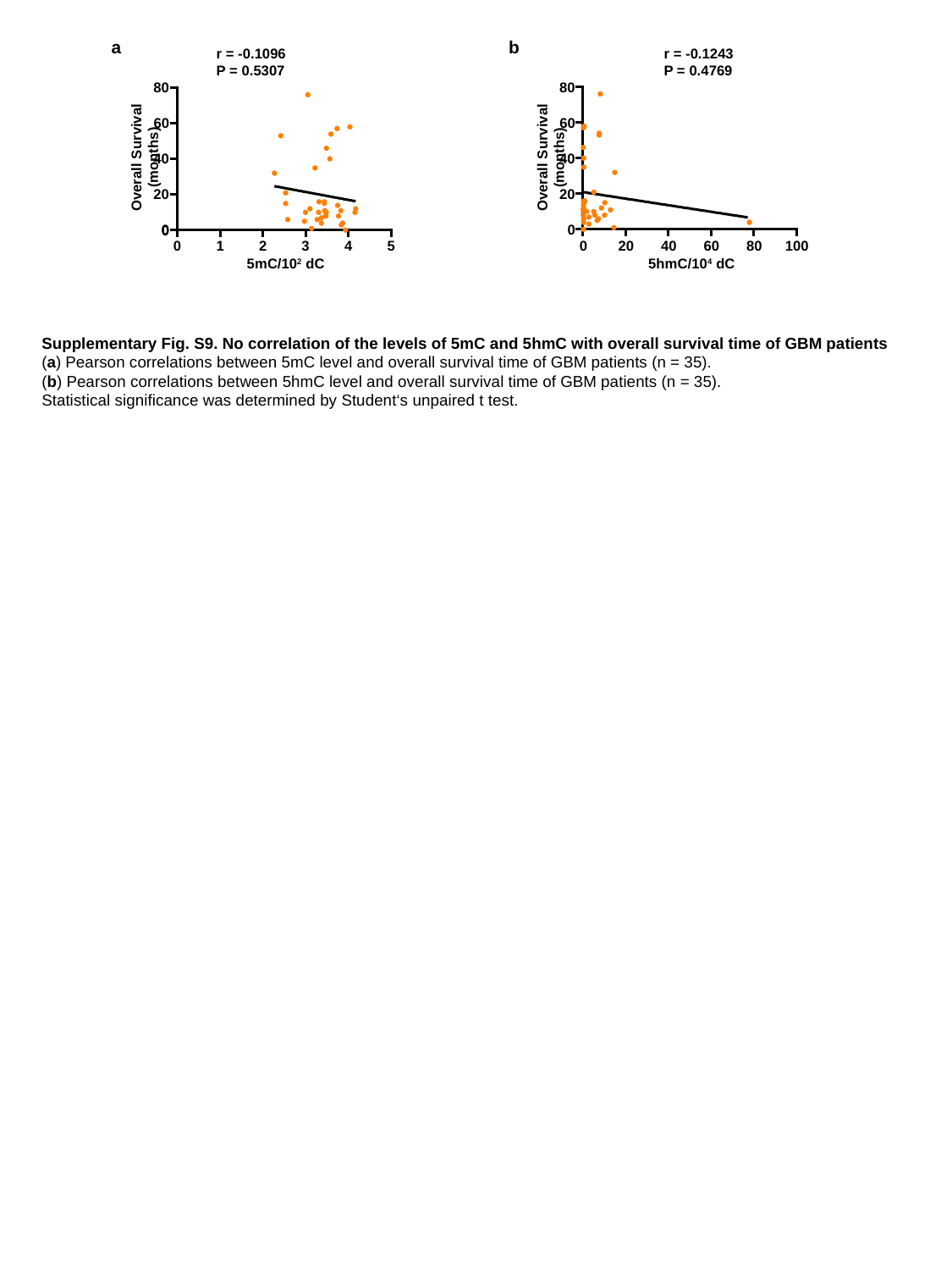

a
b
r = -0.1096
P = 0.5307
r = -0.1243
P = 0.4769
80
60
40
20
0
Overall Survival (months)
3
0
1
2
4
5
5mC/102 dC
80
60
40
20
0
Overall Survival (months)
60
0
20
40
80
100
5hmC/104 dC
0
Supplementary Fig. S9. No correlation of the levels of 5mC and 5hmC with overall survival time of GBM patients
(a) Pearson correlations between 5mC level and overall survival time of GBM patients (n = 35).
(b) Pearson correlations between 5hmC level and overall survival time of GBM patients (n = 35).
Statistical significance was determined by Student‘s unpaired t test.

## Slide 11
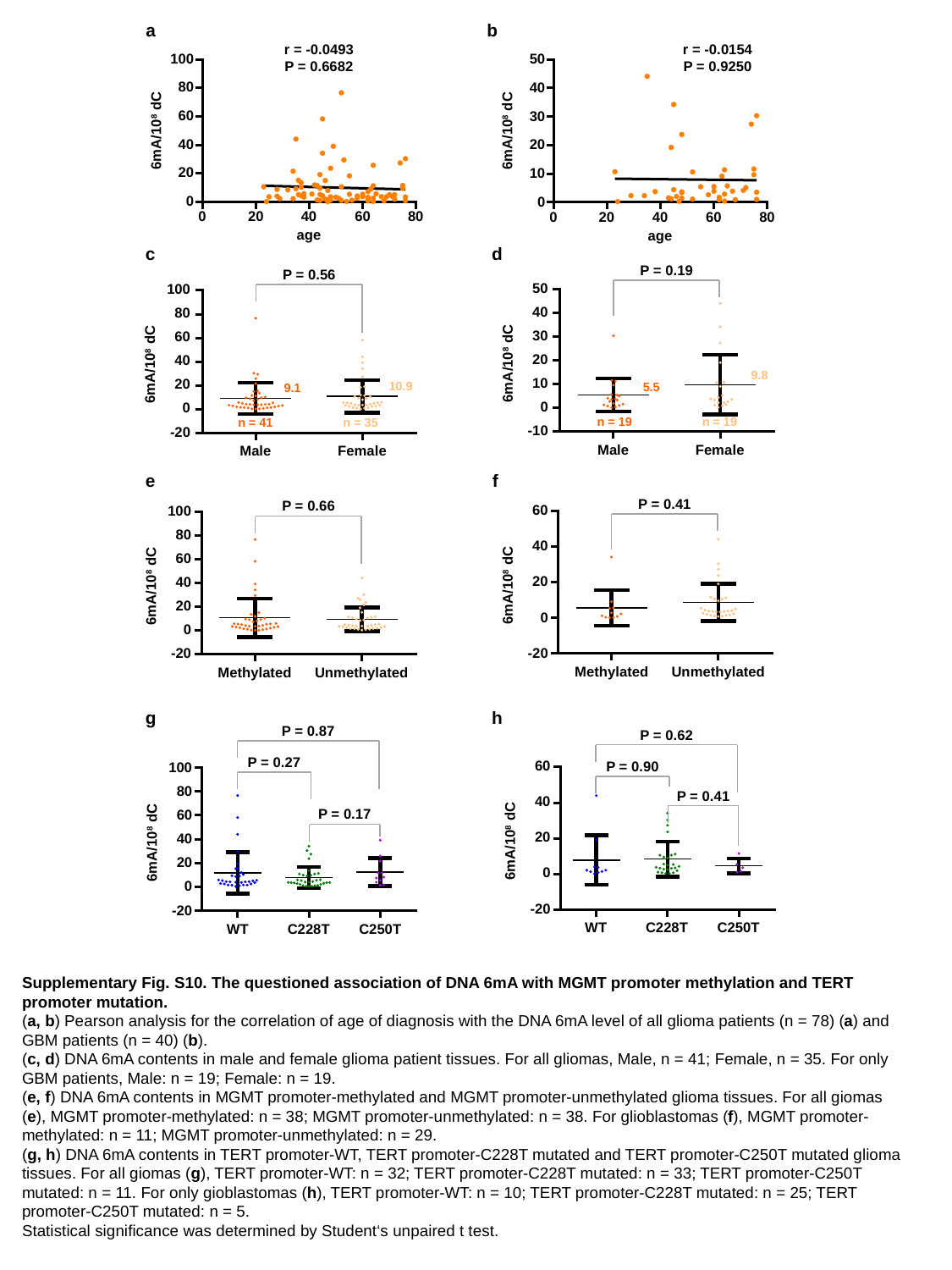

a
b
100
80
60
40
20
0
6mA/108 dC
60
0
20
40
80
age
50
40
30
20
10
0
6mA/108 dC
60
0
20
40
80
age
r = -0.0493
P = 0.6682
r = -0.0154
P = 0.9250
c
d
P = 0.19
50
40
30
20
10
0
-10
6mA/108 dC
Male
Female
9.8
5.5
n = 19
n = 19
P = 0.56
100
80
60
40
20
0
-20
6mA/108 dC
Male
Female
10.9
9.1
n = 41
n = 35
e
f
P = 0.41
60
40
20
0
-20
6mA/108 dC
Methylated
Unmethylated
P = 0.66
100
80
60
40
20
0
-20
6mA/108 dC
Methylated
Unmethylated
g
h
P = 0.87
P = 0.27
P = 0.17
P = 0.62
P = 0.90
P = 0.41
60
40
20
0
-20
6mA/108 dC
WT
C228T
C250T
100
80
60
40
20
0
-20
6mA/108 dC
WT
C228T
C250T
Supplementary Fig. S10. The questioned association of DNA 6mA with MGMT promoter methylation and TERT promoter mutation.
(a, b) Pearson analysis for the correlation of age of diagnosis with the DNA 6mA level of all glioma patients (n = 78) (a) and GBM patients (n = 40) (b).
(c, d) DNA 6mA contents in male and female glioma patient tissues. For all gliomas, Male, n = 41; Female, n = 35. For only GBM patients, Male: n = 19; Female: n = 19.
(e, f) DNA 6mA contents in MGMT promoter-methylated and MGMT promoter-unmethylated glioma tissues. For all giomas (e), MGMT promoter-methylated: n = 38; MGMT promoter-unmethylated: n = 38. For glioblastomas (f), MGMT promoter-methylated: n = 11; MGMT promoter-unmethylated: n = 29.
(g, h) DNA 6mA contents in TERT promoter-WT, TERT promoter-C228T mutated and TERT promoter-C250T mutated glioma tissues. For all giomas (g), TERT promoter-WT: n = 32; TERT promoter-C228T mutated: n = 33; TERT promoter-C250T mutated: n = 11. For only gioblastomas (h), TERT promoter-WT: n = 10; TERT promoter-C228T mutated: n = 25; TERT promoter-C250T mutated: n = 5.
Statistical significance was determined by Student‘s unpaired t test.

## Slide 12
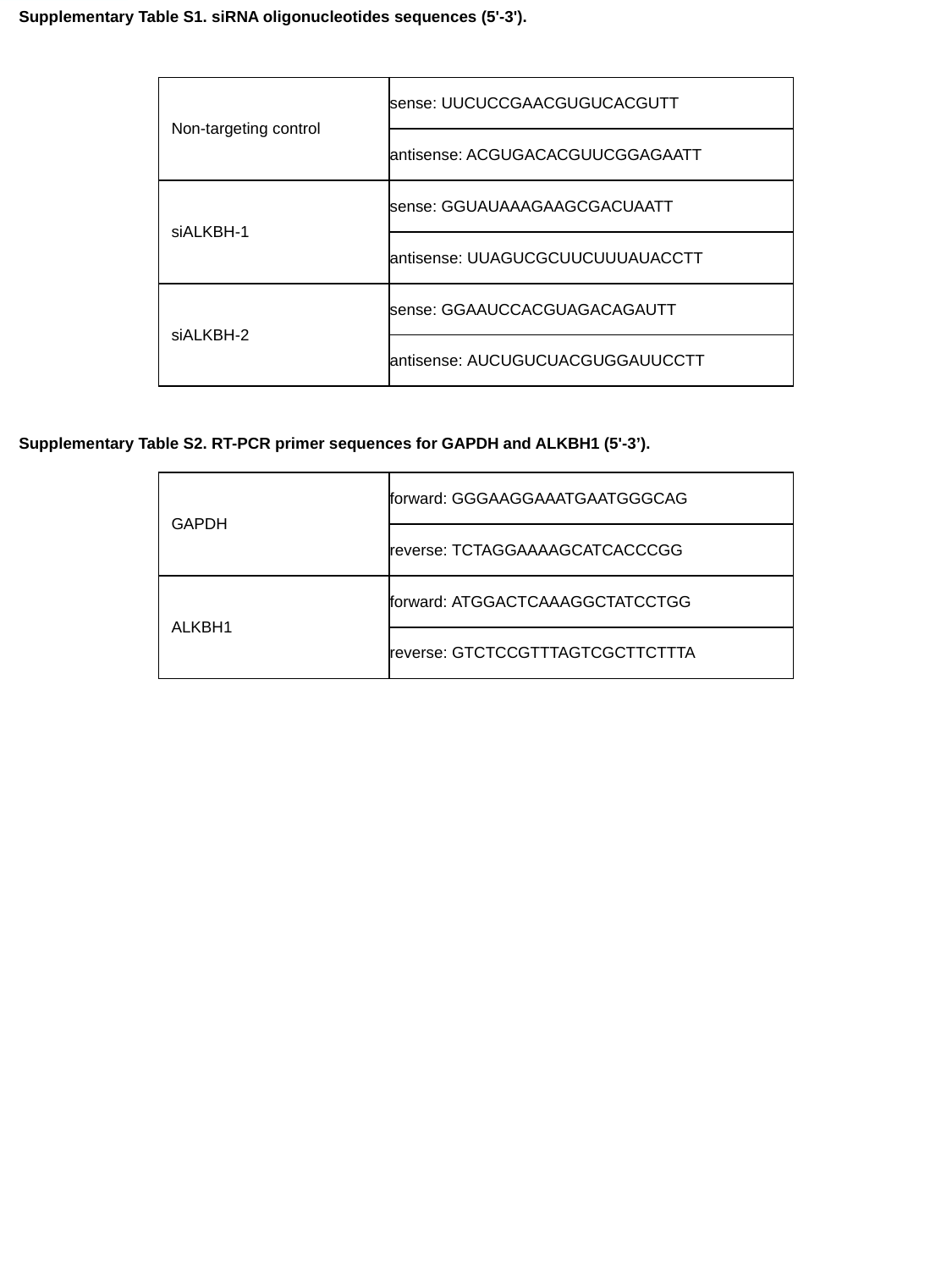

Supplementary Table S1. siRNA oligonucleotides sequences (5'-3').
| Non-targeting control | sense: UUCUCCGAACGUGUCACGUTT |
| --- | --- |
| Non-targeting control | antisense: ACGUGACACGUUCGGAGAATT |
| siALKBH-1 | sense: GGUAUAAAGAAGCGACUAATT |
| | antisense: UUAGUCGCUUCUUUAUACCTT |
| siALKBH-2 | sense: GGAAUCCACGUAGACAGAUTT |
| | antisense: AUCUGUCUACGUGGAUUCCTT |
Supplementary Table S2. RT-PCR primer sequences for GAPDH and ALKBH1 (5'-3’).
| GAPDH | forward: GGGAAGGAAATGAATGGGCAG |
| --- | --- |
| Non-targeting control | reverse: TCTAGGAAAAGCATCACCCGG |
| ALKBH1 | forward: ATGGACTCAAAGGCTATCCTGG |
| | reverse: GTCTCCGTTTAGTCGCTTCTTTA |

## Slide 13
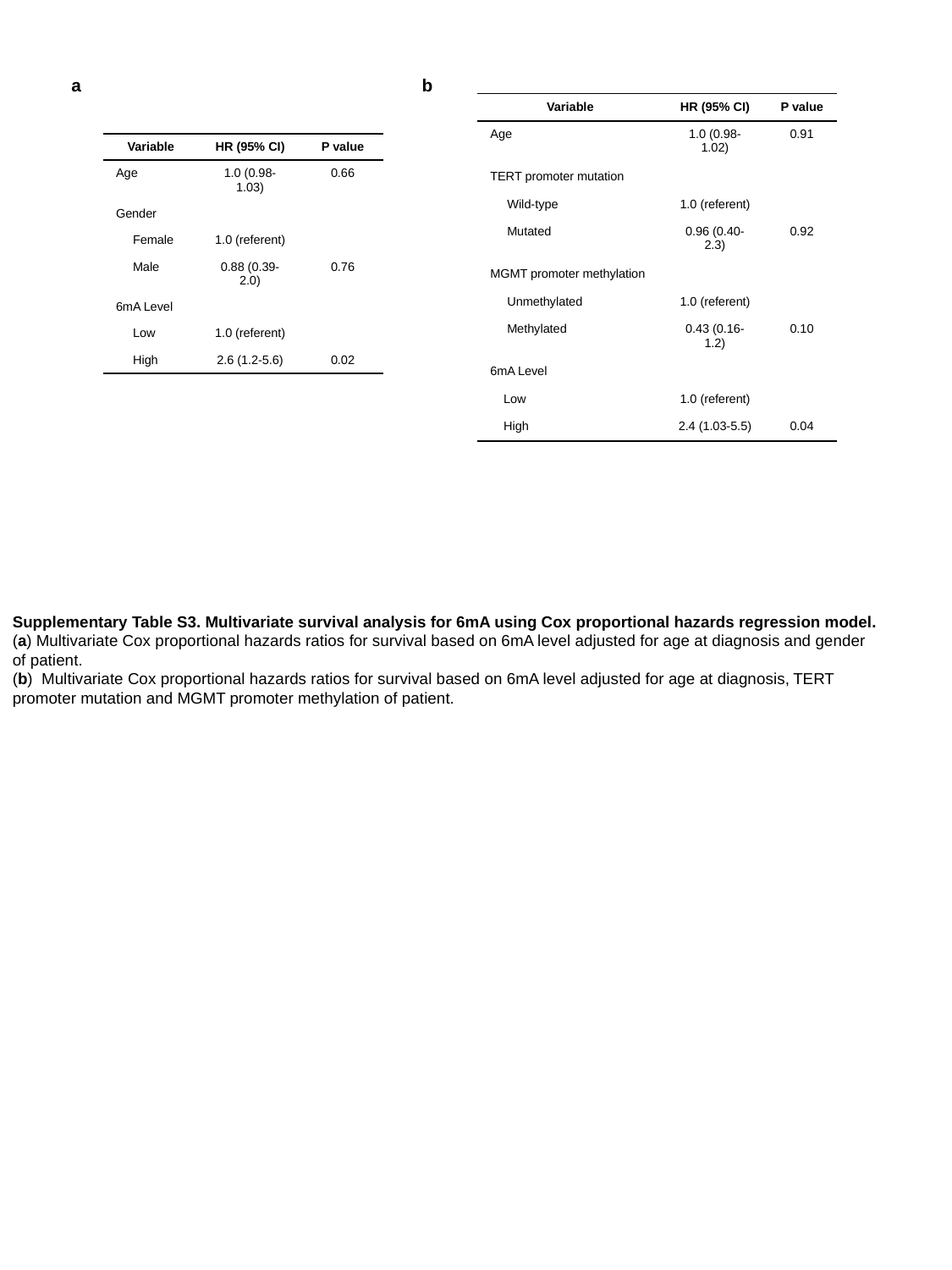

a
b
| Variable | HR (95% CI) | P value |
| --- | --- | --- |
| Age | 1.0 (0.98-1.02) | 0.91 |
| TERT promoter mutation | | |
| Wild-type | 1.0 (referent) | |
| Mutated | 0.96 (0.40-2.3) | 0.92 |
| MGMT promoter methylation | | |
| Unmethylated | 1.0 (referent) | |
| Methylated | 0.43 (0.16-1.2) | 0.10 |
| 6mA Level | | |
| Low | 1.0 (referent) | |
| High | 2.4 (1.03-5.5) | 0.04 |
| Variable | HR (95% CI) | P value |
| --- | --- | --- |
| Age | 1.0 (0.98-1.03) | 0.66 |
| Gender | | |
| Female | 1.0 (referent) | |
| Male | 0.88 (0.39-2.0) | 0.76 |
| 6mA Level | | |
| Low | 1.0 (referent) | |
| High | 2.6 (1.2-5.6) | 0.02 |
Supplementary Table S3. Multivariate survival analysis for 6mA using Cox proportional hazards regression model.
(a) Multivariate Cox proportional hazards ratios for survival based on 6mA level adjusted for age at diagnosis and gender of patient.
(b) Multivariate Cox proportional hazards ratios for survival based on 6mA level adjusted for age at diagnosis, TERT promoter mutation and MGMT promoter methylation of patient.

## Slide 14
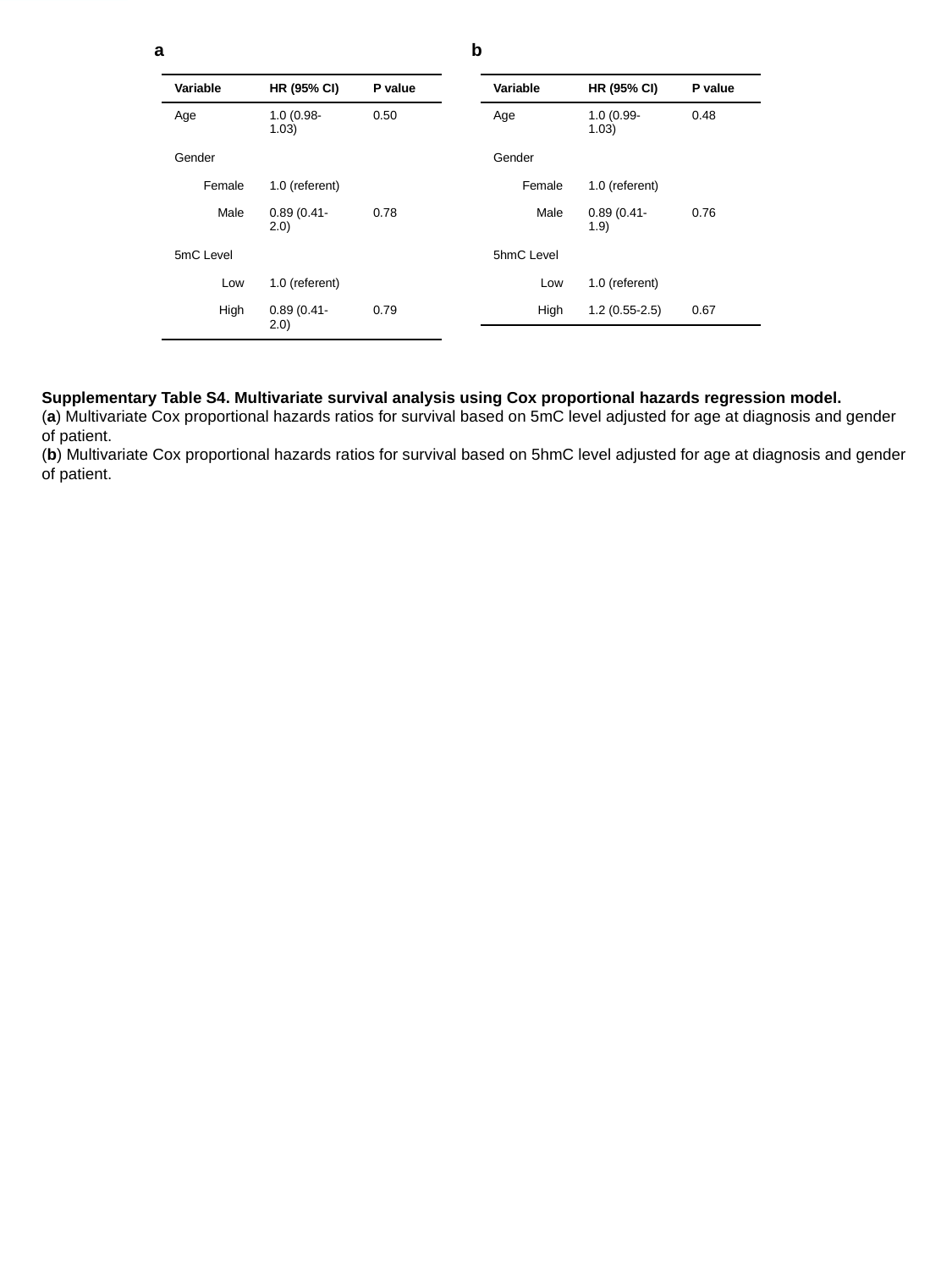

a
b
| Variable | HR (95% CI) | P value |
| --- | --- | --- |
| Age | 1.0 (0.98-1.03) | 0.50 |
| Gender | | |
| Female | 1.0 (referent) | |
| Male | 0.89 (0.41-2.0) | 0.78 |
| 5mC Level | | |
| Low | 1.0 (referent) | |
| High | 0.89 (0.41-2.0) | 0.79 |
| Variable | HR (95% CI) | P value |
| --- | --- | --- |
| Age | 1.0 (0.99-1.03) | 0.48 |
| Gender | | |
| Female | 1.0 (referent) | |
| Male | 0.89 (0.41-1.9) | 0.76 |
| 5hmC Level | | |
| Low | 1.0 (referent) | |
| High | 1.2 (0.55-2.5) | 0.67 |
Supplementary Table S4. Multivariate survival analysis using Cox proportional hazards regression model.
(a) Multivariate Cox proportional hazards ratios for survival based on 5mC level adjusted for age at diagnosis and gender of patient.
(b) Multivariate Cox proportional hazards ratios for survival based on 5hmC level adjusted for age at diagnosis and gender of patient.
